# Atypical antidepressants extend lifespan of *Caenorhabditis elegans* by activation of a non‐cell‐autonomous stress response

**DOI:** 10.1111/acel.12379

**Published:** 2015-08-08

**Authors:** Sunitha Rangaraju, Gregory M. Solis, Sofia I. Andersson, Rafael L. Gomez‐Amaro, Rozina Kardakaris, Caroline D. Broaddus, Alexander B. Niculescu, Michael Petrascheck

**Affiliations:** ^1^ Department of Chemical PhysiologyThe Scripps Research InstituteLa JollaCAUSA; ^2^ Department of Molecular and Experimental MedicineThe Scripps Research InstituteLa JollaCAUSA; ^3^ Department of Molecular and Cellular NeuroscienceThe Scripps Research InstituteLa JollaCAUSA; ^4^ Department of PsychiatryIndiana University School of MedicineIndianapolisINUSA

**Keywords:** antidepressant, anti‐aging, *Caenorhabditis elegans*, non‐cell‐autonomous, psychiatric disease, signal transduction, synaptic transmission, stress

## Abstract

Oxidative stress has long been associated with aging and has recently been linked to psychiatric disorders, including psychosis and depression. We identified multiple antipsychotics and antidepressants that extend *Caenorhabditis elegans* lifespan and protect the animal from oxidative stress. Here, we report that atypical antidepressants activate a neuronal mechanism that regulates the response to oxidative stress throughout the animal. While the activation of the oxidative stress response by atypical antidepressants depends on synaptic transmission, the activation by reactive oxygen species does not. Lifespan extension by atypical antidepressants depends on the neuronal oxidative stress response activation mechanism. Neuronal regulation of the oxidative stress response is likely to have evolved as a survival mechanism to protect the organism from oxidative stress, upon detection of adverse or dangerous conditions by the nervous system.

## Introduction

In contrast to aging, where the role of oxidative stress has been de‐emphasized in recent years, oxidative stress has been gaining increased attention as a pathogenic mechanism underlying several major psychiatric disorders, including psychosis and depression (Scapagnini *et al*., 2012; Pandya *et al*., 2013). Behavioral paradigms in rodents that are depressogenic (e.g., restraint stress, forced swim test) increase lipid peroxidation in several tissues (Sahin *et al*., 2004; Lucca *et al*., 2009; Runkel *et al*., 2013; Spiers *et al*., 2013). Similarly, tissue samples from psychiatric patients show increased levels of lipid peroxidation and depletion of antioxidant activities compared to samples from healthy controls (Do *et al*., 2000; Bilici *et al*., 2001; Gawryluk *et al*., 2011; Ditzen *et al*., 2012). These effects on oxidative defense systems may be more than just an indirect correlation, as suggested by experiments showing that certain antioxidants have antidepressant‐like effects in rodents as well as in humans (Ferreira *et al*., 2008).

In a previous study, we screened for compounds that extend lifespan in the nematode *Caenorhabditis elegans* and identified 57 new compounds, 33 of which also increased resistance to oxidative stress. Among the compounds that increased both *C. elegans* lifespan and resistance to oxidative stress were 6 clinically approved antidepressants and antipsychotics. These drugs are used to treat mental disorders, which have been associated with stress in general and, as mentioned above, with oxidative stress in specific (Petrascheck *et al*., 2007; Ye *et al*., 2014).

In this study, we set out to investigate the mechanism by which different antidepressants modulate resistance to oxidative stress. As antidepressants exert their therapeutic effect by acting on the nervous system, we asked whether these drugs protect *C. elegans* by a similar mechanism from oxidative stress. To test this prediction, we studied how resistance to oxidative stress and lifespan changed in response to antidepressant treatment and how these changes are affected by mutations in oxidative stress response genes and genes controlling synaptic transmission.

## Results

### Atypical antidepressants protect *C. elegans* from oxidative stress

**Figure 1:**
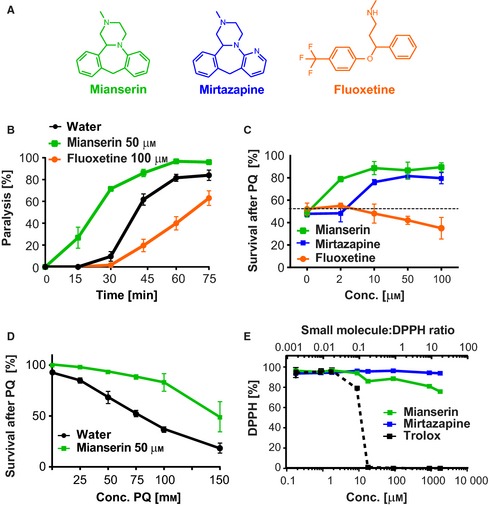
The atypical antidepressant Mianserin increases synaptic transmission and resistance to oxidative stress. (A) Structures of the atypical antidepressants Mianserin and Mirtazapine and the typical antidepressant Fluoxetine. (B) Mianserin increases and Fluoxetine decreases synaptic transmission as measured by aldicarb‐induced paralysis. Wild‐type L4‐stage animals were pretreated with 50 μm Mianserin or 100 μm Fluoxetine for 2 h, followed by 4 mm of the acetylcholine esterase inhibitor aldicarb. Aldicarb‐induced paralysis, a measure of synaptic transmission, was determined every 15 min and plotted in [%] (*y*‐axis) as a function of time in minutes [min] (*x*‐axis). (C) Antidepressant treatment affects the survival of *Caenorhabditis elegans* under conditions of oxidative stress. Wild‐type day 1 adults were treated with increasing concentrations of the indicated antidepressants, followed by 100 mm of the ROS generator paraquat on day 5. Survival of animals was determined 24 h later and plotted in [%] (*y*‐axis) as a function of antidepressant concentration [μm] (*x*‐axis). Dotted line shows the survival of untreated animals. (D) Mianserin treatment protects *C. elegans* from a range of paraquat concentrations. Wild‐type day 1 adults were treated with water or 50 μm Mianserin, followed by increasing concentrations of paraquat on day 5. Survival of animals was determined 24 h later and plotted in [%] (*y*‐axis) as a function of paraquat concentration [mm] ((*x*‐axis). (E) Antidepressants have no significant free radical‐scavenging activity. Increasing concentrations of either the indicated antidepressants or the ROS scavenger Trolox were incubated with 90 μm of the free radical DPPH. Reduction of DPPH [%] was monitored by measuring absorption at 520 nm (*y*‐axis) and plotted as the ratio of small molecule to DPPH (upper *x*‐axis) or the concentration of small molecule in μm (lower *x*‐axis). All error bars show SEM. for multiple, independent experiments. For additional data, see Fig. S1 (Supporting information). For detailed statistics, see Tables S1–S3 (Supporting information).

Based on the results of our previous study, we set out to investigate the ability of different antidepressant mechanisms to protect *C. elegans* from oxidative stress caused by the reactive oxygen species (ROS) generator paraquat. We chose to investigate the effects of the *atypical* antidepressants Mianserin and Mirtazapine, two structurally related antidepressants, and the *typical* antidepressant Fluoxetine (Fig. 1A). The *typical* antidepressant Fluoxetine, better known under its brand name Prozac, inhibits re‐uptake of serotonin from the synaptic cleft into the presynaptic neuron, thereby enhancing serotonergic signaling. In contrast, *atypical* antidepressants like Mianserin and Mirtazapine antagonize serotonin and noradrenalin receptors, thereby inhibiting serotonergic signaling (Petrascheck *et al*., 2007). Despite their seemingly opposing effects on serotonergic signaling, all three drugs act as antidepressants in humans.

First, we assessed the effect of the atypical antidepressant Mianserin and typical antidepressant Fluoxetine on the *C. elegans* nervous system. We compared the effects of Mianserin and Fluoxetine on synaptic neurotransmitter release in *C. elegans* by determining the onset of paralysis induced by the acetylcholine esterase inhibitor aldicarb (Fig. 1B). Aldicarb paralyzes the animal through hypercontraction of body wall muscles, achieved by preventing the breakdown of acetylcholine at the neuromuscular junction, thereby enhancing cholinergic signaling. Mianserin treatment caused animals to become hypersensitive to aldicarb‐induced paralysis implying enhanced synaptic release of acetylcholine, while Fluoxetine treatment caused animals to become resistant implying decreased synaptic release of acetylcholine (Fig. 1B, Table S1). These results confirm the opposing effects of Mianserin and Fluoxetine on synaptic release of neurotransmitters, with Mianserin increasing and Fluoxetine decreasing the release (Nurrish *et al*., 1999; Kullyev *et al*., 2010).

We next compared the ability of these three antidepressants to protect from oxidative stress by treating day 1 adults with increasing concentrations of each antidepressant (Fig. 1C). After 5 days of treatment, the animals were subjected to the ROS generator paraquat (100 mm) (Runkel *et al*., 2013). Survival was determined 24 hours (h) later. Mianserin and Mirtazapine markedly protected the animals from oxidative stress and hence increased survival in a dose‐dependent manner (Fig. 1C, Table S2). In contrast, Fluoxetine did not protect from oxidative stress, and at higher concentrations, it decreased survival (Fig. 1C, Table S2). The protective effect of Mianserin was observed over a wide range of paraquat concentrations (Fig. 1D, Tables S3 and S4). We concluded that the *atypical* antidepressants Mianserin and Mirtazapine protect from oxidative stress, while the *typical* antidepressant Fluoxetine does not.

We next asked whether Mianserin and Mirtazapine protect from oxidative stress by scavenging ROS (Fig. 1E). Free radical‐scavenging activity of a compound *in vitro* can be monitored using the free radical DPPH that absorbs light at 520 nm, while its reduced form does not (Pisoschi *et al*., 2009). Neither Mirtazapine nor Mianserin showed a DPPH‐scavenging activity at any relevant concentration. Scavenging activity of Mianserin was only observed at a ratio of 25:1 (Mia: DPPH), which is 50,000 times higher than what was used in the *in vivo* stress resistance assays (Mia: PQ; 1:2000) (Fig. 1E). Therefore, we deemed ROS scavenging to be an unlikely mechanism for these antidepressants to protect *C. elegans* from oxidative stress.

Earlier studies have shown that paraquat blocks the development of *C. elegans* L1 larvae by entering the body through a specialized channel, formed by bundles of amphid dendrites, designed to sample fluids in the environment. This channel is absent in dye‐filling mutants (*dyf*), such as *osm‐3(p802)* that lack amphid neurons and, as a result, are resistant to the inhibitory effect of paraquat on development (Fujii *et al*., 2004). We confirmed the resistance of *osm‐3(p802)* larvae to the paraquat‐induced block in development (Fig. S1A). However, adult *osm‐3(p802)* animals showed no increase in paraquat resistance compared to wild‐type N2 animals (Fig. S1B), suggesting that the regulation of resistance to oxidative stress differs between larvae and adults.

Taken together, these results show that the *atypical* antidepressant Mianserin increases synaptic transmission and protection from oxidative stress, while the *typical* antidepressant Fluoxetine decreases synaptic transmission and does not protect from oxidative stress.

### Mianserin‐induced protection from oxidative stress requires SOD‐1, CTL‐1, and PRDX‐2

**Figure 2:**
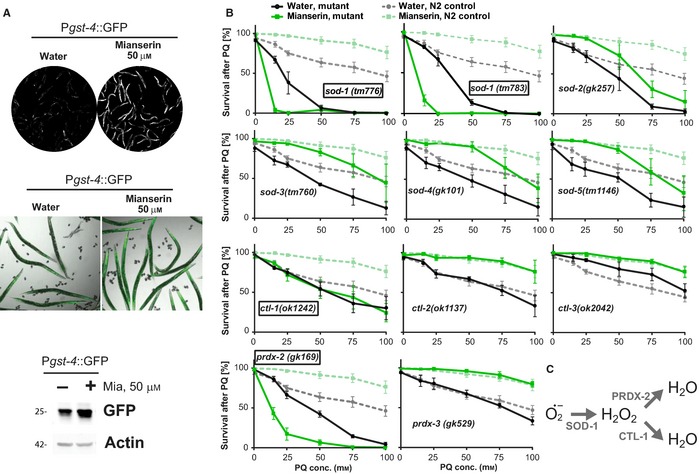
Mianserin activates a protective oxidative stress response. (A) Mianserin induces expression of the P*gst‐4*::GFP reporter. Images show GFP fluorescence of water‐ or Mianserin‐treated (50 μm) reporter animals after 24‐h treatment. Images were taken using identical exposure settings (upper panel). Merged image of bright‐field and GFP fluorescence is shown (middle panel). Localization of GFP in water‐treated worms and induced GFP in Mianserin‐treated worms can be visualized. Verification of *Pgst‐4*::GFP induction by immunoblotting on day 5 (lower panel). Immunoblot of water‐ or Mianserin‐treated (50 μm) *Pgst‐4*::GFP worm lysates, probed with GFP or actin (loading control) antibodies. (B) Mianserin‐induced protection from oxidative stress requires specific oxidative stress response genes. Wild‐type or mutant day 1 adults were treated with water or 50 μm Mianserin, followed by increasing concentrations of paraquat on day 5. Survival of animals was determined 24 h later and plotted in [%] (*y*‐axis) as a function of paraquat concentration [mm] (*x*‐axis). Parallel wild‐type (N2) control experiments (dotted lines) are shown for each graph. Genotypes (indicated in each graph) for which Mianserin failed to increase resistance to oxidative stress are boxed. (C) Mianserin‐induced superoxide detoxification pathway. Superoxides (O_2_*^−^) are converted to H_2_O_2_ by SOD‐1, followed by a conversion to water by PRDX‐2 and CTL‐1. All error bars show S.E.M. for 3 or more independent experiments. For additional data, see Figures S2 and S3 (Supporting information). For detailed statistics, see Table S3 (Supporting information).

Because Mianserin treatment protects *C. elegans* from the superoxide generator paraquat (Fig. 1), we next focused on genes involved in the superoxide defense mechanism (Fig. 2). Superoxides are converted to hydrogen peroxide by superoxide dismutases (SOD) followed by a conversion to water by catalases (CTL) or peroxiredoxins (PRDX) (Sies, 2014). The *C. elegans* genome encodes 5 different superoxide dismutases (*sod‐1* to *sod‐5*), three different catalases (*ctl‐1* to *ctl‐3*), and three peroxiredoxins that are named according to their homology to the mammalian peroxiredoxins, *prdx‐2*,* prdx‐3,* and *prdx‐6* (Doonan *et al*., 2008; Olahova *et al*., 2008; Kumsta *et al*., 2011).

Activation of the oxidative stress response by Mianserin was confirmed through fluorescent imaging on day 2, as well as immunoblotting on day 5 using a reporter strain in which GFP transcription is driven by the glutathione s‐transferase‐4 promoter (P*gst‐4*::GFP) (Leiers *et al*., 2003) (Fig. 2A). While a 2‐h Mianserin treatment increased synaptic transmission (Fig. 1B), it did not induce the P*gst‐4*::GFP reporter (Fig. S2). To determine which genes are necessary for Mianserin to protect from oxidative stress, we treated mutants lacking individual *sod*,* ctl,* or *prdx* genes with Mianserin or water and subjected them to increasing concentrations of paraquat (Fig. 2B). As previously seen, mutations in many of the *sod*,* ctl,* or *prdx* genes decreased the survival of water‐treated animals (Fig. 2B, Table S3), confirming the requirement of these genes for wild‐type resistance to oxidative stress (Van Raamsdonk & Hekimi, 2009). In contrast, Mianserin protected most mutants from oxidative stress, except *sod‐1(tm776), sod‐1(tm783), ctl‐1(ok1242),* and *prdx‐2(gk169)* animals (Fig. 2B, Table S3).

Surprisingly, *sod‐3* was not required for the induction of stress resistance by Mianserin (Fig. 2B). *sod‐3* encodes a mitochondrial superoxide dismutase that is widely used as an oxidative stress reporter and that is activated by the FOXO transcription factor DAF‐16 in response to decreased insulin signaling and other aging pathways. The observation that *sod‐3* is dispensable for the Mianserin‐induced stress response implies that the Mianserin‐induced stress response is independent of DAF‐16. This prediction was confirmed as Mianserin increased the stress resistance in *daf‐16(mu86)* animals to the same extent as it did in N2 animals (Fig. S3A). Mianserin did not further increase the stress resistance in the gain‐of‐function mutant *skn‐1(lax188)* (Paek *et al*., 2012) that is already highly resistant to oxidative stress (Fig. S3B), suggesting SKN‐1 is involved in the protective mechanism. Taken together, these results show that Mianserin activates a protective mechanism by which SOD‐1 converts superoxide radicals to hydrogen peroxide and CTL‐1 and PRDX‐2 convert hydrogen peroxide to water (Fig. 2C).

### Mianserin‐induced regulation of the oxidative stress response is dependent on synaptic transmission

**Figure 3:**
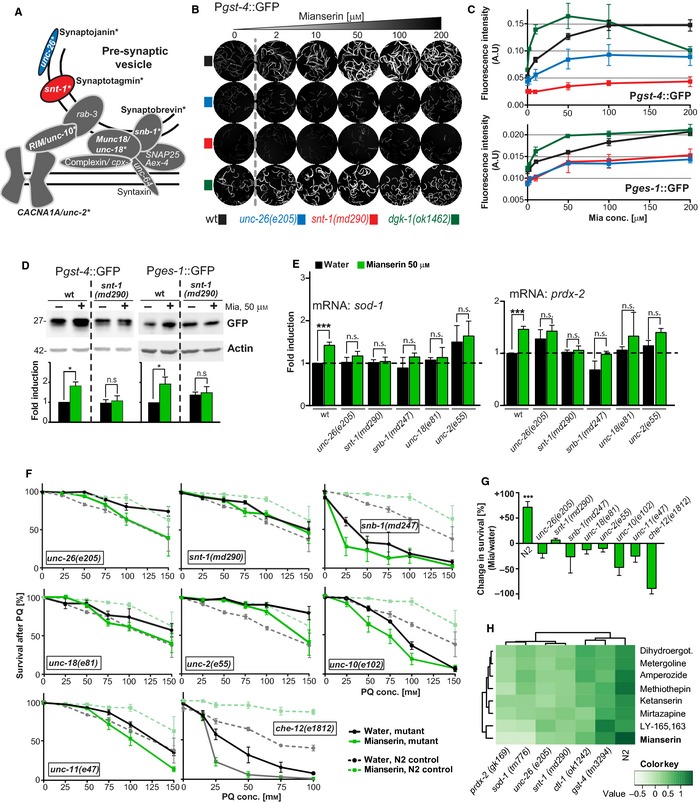
Mianserin‐mediated activation of the oxidative stress response is dependent on synaptic transmission and chemosensation. (A) Cartoon of a presynaptic vesicle compartment and physical localization of synaptic components. Synaptic components are labeled using mammalian as well as *Caenorhabditis elegans* nomenclature. Synaptic components tested in this study, namely *unc‐26, snt‐1, snb‐1, unc‐18*,* unc‐2, and unc‐10,* are highlighted in bold and marked with asterisks. Synaptojanin (UNC‐26, blue) and synaptotagmin (SNT‐1, red) are color‐coded. (B) Mianserin induces expression of the *Pgst‐4::* GFP reporter via synaptic transmission. Images show GFP fluorescence of reporter animals. Day 1 adult wild‐type or synaptic mutant *Pgst‐4::* GFP reporter animals were treated with increasing concentrations of Mianserin and fluorescent images were taken 24 h later. Reduced synaptic transmission *(unc‐26(e205)* or *snt‐1(md290*)) reduces the induction of the *Pgst‐4::* GFP reporter by Mianserin. Enhanced synaptic transmission *(dgk‐1(ok1462))* (dark green) enhances the induction of the *Pgst‐4::* GFP reporter by Mianserin. (C) Quantification of Mianserin‐induced fluorescence of strains carrying P*gst‐4::* GFP (upper panel) and P*ges‐1::* GFP reporters (lower panel). Images are not shown for *ges‐1::* GFP. Fluorescent intensity arbitrary units [A.U.] (*y*‐axis) is plotted as a function of Mianserin concentration [μm] (*x*‐axis). (D) Immunoblot verification of *Pgst‐4*::GFP induction by Mianserin on day 5 in wild‐type or *snt‐1(md290)* strains carrying the P*gst‐4::* GFP or P*ges‐1*::GFP reporter. Animals were treated with water or Mianserin (50 μm) on day 1 and harvested on day 5, followed by lysate preparation and probing with GFP or actin (loading control) antibodies. (E) Mianserin fails to induce transcription of *sod‐1* and *prdx‐2* in animals with impaired synaptic transmission. Wild‐type (N2) or mutant day 1 adults were treated with water or 50 μm Mianserin, and RNA was harvested on day 5. Gene expression levels were measured by qRT‐PCR and plotted as fold induction (*y*‐axis) over transcript levels of untreated, wild‐type animals. (F) Mianserin‐induced protection from oxidative stress requires synaptic transmission and chemosensory neuron function. Wild‐type or mutant day 1 adults were treated with water or 50 μm Mianserin, followed by increasing concentrations of paraquat on day 5. Survival of animals was determined 24 h later and plotted in [%] (*y*‐axis) as a function of paraquat concentration [mm] (*x*‐axis). Parallel wild‐type (N2) control experiments (dotted lines) are shown for each graph. Genotypes (indicated in each graph) for which Mianserin failed to increase resistance to oxidative stress are boxed. (G) Percent change in survival of Mianserin‐treated compared to water‐treated animals after 24‐h PQ treatment (100 mm). *Y*‐axis shows fold change in survival [%] (Mianserin/water). (H) Hierarchical clustering of fold change in paraquat protection of wt and mutant animals shows the degree of similarity between genotypes (top) or eight structurally distinct serotonin antagonists (left). (Dihydroergot stands for dihydroergotamine.) All error bars show SEM of 3–11 independent experiments. For detailed statistics, see Tables S4 and S5 (Supporting information). ***P < 0.001, n.s.: not significant, *P* > 0.05.

In humans, antidepressants such as Mianserin exert their therapeutic effects by acting on the nervous system. We therefore asked whether the stress‐protective effect of Mianserin depends on the drug's ability to enhance synaptic transmission (Fig. 1B). To test this, we used strains with impaired synaptic transmission caused by mutations in synaptic genes such as *unc‐2*/CACNA1A, *unc‐18*/Munc18, *snt‐1*/synaptotagmin, *snb‐1*/synaptobrevin, *unc‐26*/synaptojanin, *unc‐10*/Rim1, and *unc‐11*/AP180 (Fig. 3A) (Barclay *et al*., 2012; Bargmann, 2012). We first focused on the calcium sensor synaptotagmin (SNT‐1), required for Ca^2+^‐induced vesicle exocytosis (Brose *et al*., 1992), and synaptojanin (UNC‐26), required for vesicle recycling (Harris *et al*., 2000). Mutations in these genes decrease synaptic transmission (Nonet *et al*., 1993; Harris *et al*., 2000). In contrast, diacylglycerol kinase‐1 (DGK‐1) represses synaptic exocytosis, and therefore, mutations in *dgk‐1* enhance synaptic transmission (Nurrish *et al*., 1999). Mutations in *unc‐26* and *dgk‐1* have been shown to exert opposing effects on size and spatial localization of synaptic vesicles confirming their opposing roles for synaptic vesicle function (Nurrish *et al*., 1999; Ch'ng *et al*., 2008).

To monitor the effects of altered synaptic transmission on the expression of oxidative stress genes (Fig. 3B), we crossed animals with decreased (*unc‐26, snt‐1*) or enhanced (*dgk‐1*) synaptic transmission with the reporter strain carrying the P*gst‐4*::GFP transgene (Leiers *et al*., 2003). In wild‐type animals, a 24‐h Mianserin treatment increased the expression of the oxidative stress reporter P*gst‐4*::GFP in a dose‐dependent manner (Fig. 3B,C). Mutations that decrease synaptic transmission, such as *unc‐26(e205)* and *snt‐1(md290),* blunted or abolished the Mianserin‐induced expression of P*gst‐4*::GFP. In contrast, mutations that enhance synaptic transmission, like *dgk‐1(ok1462)*, enhanced the Mianserin‐induced expression of P*gst‐4*::GFP to an even greater extent than what was observed in wild‐type animals (Fig. 3B,C). Consistent with the idea of synaptic transmission modulating the oxidative stress response, even in the absence of Mianserin, baseline P*gst‐4*::GFP expression was slightly higher in animals with enhanced synaptic transmission (*dgk‐1(ok1462))* and lower in the synaptic mutants with diminished synaptic transmission *(unc‐26(e205)* and *snt‐1(md290))* (Fig. 3B,C). We chose *dgk‐1* because it showed an inverse correlation with *unc‐26* in synaptic vesicle dynamics (Ch'ng *et al*., 2008), which is also reflected in our results using the P*gst‐4*::GFP reporter (Fig. 3B). In addition, *dgk‐1* is involved in serotonergic signaling, one of the major signaling pathways targeted by antidepressants such as Mianserin (Nurrish *et al*., 1999). However, *dgk‐1* is not exclusively expressed in the nervous system but is also expressed in excretory canals (Nurrish *et al*., 1999). Thus, to exclude the involvement of other tissues, testing additional mutants with enhanced synaptic transmission such a *cpx‐1(ok1552)* or *tomo‐1(nu468)* is warranted (Hobson *et al*., 2011; Chan *et al*., 2012).

We further found that Mianserin activates the expression of the type B carboxylesterase *ges‐1* (Fig. 3C, lower panel), a gene exclusively expressed in the gut (Benedetti *et al*., 2006). As with the P*gst‐4*::GFP reporter, Mianserin dose‐dependently induced the expression of P*ges‐1*::GFP in a manner dependent on synaptic transmission. Similar to the case with P*gst‐4*::GFP, in animals with reduced synaptic transmission like *snt‐1(md290)* and *unc‐26(e205)* mutants, the P*ges‐1*::GFP induction was blunted, while it was enhanced in *dgk‐1(ok1462)* mutants (Fig. 3C). The sustained activation of P*gst‐4*::GFP on day 5 was confirmed using immunoblotting (Fig. 3D). Therefore, Mianserin non‐cell‐autonomously activates *ges‐1* expression in the gut via synaptic transmission. This result is all the more surprising because Mianserin is likely to enter the animal through the gut and does not directly activate *ges‐1,* but instead depends on synaptic transmission. These results suggest that Mianserin regulates the expression of oxidative stress response genes non‐cell‐autonomously by way of synaptic transmission.

Next, we asked which other redox genes are activated in response to Mianserin. In wild‐type animals, Mianserin not only activated *gst‐4* via synaptic transmission (Fig. 2A), but also activated the expression of *sod‐1* and *prdx‐2*, both necessary for the protective effect of Mianserin (Fig. 2B), measured using quantitative real‐time PCR (qRT‐PCR) (Fig. 3E). Activation of *sod‐1* and *prdx‐2* by Mianserin was abolished in multiple synaptic transmission mutants, including *unc‐26(e205)*,* snt‐1(md290)*,* snb‐1(md247)*,* unc‐18(e81),* and *unc‐2(e55)* (Fig. 3E), strains that have slowed or abolished synaptic transmission.

To determine whether synaptic transmission is necessary for Mianserin to protect from oxidative stress, we treated synaptic mutants with Mianserin and subjected them to increasing concentrations of paraquat (Fig. 3F). The resistance of untreated mutants to oxidative stress varied between the different synaptic mutants, with *snb‐1(md247)* and *unc‐10 (e102)* showing a reduced resistance to oxidative stress and all other mutants showing a wild‐type‐like or increased resistance to oxidative stress (Fig. 3F, Table S4, see Discussion). However, the ability of Mianserin to protect from oxidative stress was strictly dependent on synaptic transmission. Mutations in all seven components of the synaptic transmission machinery tested abolished the ability of Mianserin to increase the survival of animals subjected to paraquat treatment (Fig. 3F,G, Table S4). In addition to these synaptic components, Mianserin required CHE‐12 to increase resistance to oxidative stress (Fig. 3F, Table S4). CHE‐12 is necessary for chemosensation and is exclusively expressed in a subset of amphid and phasmid sensory neurons (Kaplan & Horvitz, 1993). The *che‐12(e1812)* mutant was hypersensitive to paraquat (Fig. 3F), consistent with a role for chemosensation in the oxidative stress response. While some of the synaptic mutants show expression in non‐neuronal tissues, the requirement of CHE‐12 and seven synaptic factors unequivocally links the protective effect to be of neuronal origin (Fig. 3F,G).

The dependence of Mianserin on redox and synaptic genes was not exclusive to Mianserin. We tested seven additional, structurally distinct serotonin antagonists, all of which protected from paraquat in a manner that depended on the redox genes *prdx‐2* and *sod‐1*, as well as synaptic transmission genes *unc‐26* and *snt‐1* (Fig. 3H, Table S5). While *ctl‐1* and *gst‐4* were also required for the protective effect, they were required to a lesser extent than the genes mentioned above, as deduced from the hierarchical clustering (Fig. 3H, Table S5). Our results do not distinguish between *sod‐1* and *prdx‐2* to be required in the nervous system or in the periphery. However, given that *sod‐1* and *prdx‐2* are some of the most highly expressed genes (top 10% of all genes), we consider it more likely that they are involved in the detoxification of ROS in the peripheral tissues.

Taken together, these results show that Mianserin protects *C. elegans* from oxidative stress by activating a non‐cell‐autonomous mechanism that increases the expression of oxidative stress response genes in peripheral tissues via synaptic transmission.

### ROS‐induced activation of the oxidative stress response is independent of synaptic transmission

**Figure 4:**
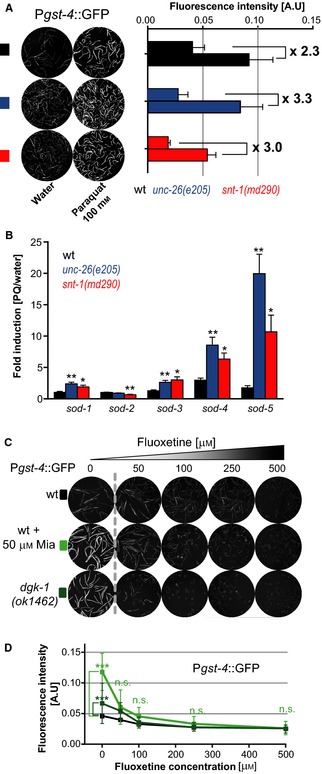
Reactive oxygen species‐mediated activation of the oxidative stress response is independent of synaptic transmission. (A) The ROS generator paraquat induces expression of the P*gst‐4::* GFP reporter independent of synaptic transmission. Images show GFP fluorescence of P*gst‐4::GFP* reporter animals. Left panel: Wild‐type or synaptic mutant P*gst‐4::* GFP reporter strains (*unc‐26(e205), snt‐1(md290))* were treated with water or 100 mm paraquat on day 1 and imaged 8 h later. Right panel: Bar graphs show quantification of fluorescent intensity [A.U.] for wild‐type or synaptic mutants. Fluorescent intensity arbitrary units [A.U.] (*y*‐axis) for water‐ and paraquat‐treated samples is indicated for each strain. (B) Paraquat induces transcription of *sod* genes in wild‐type (N2) and synaptic mutant animals. Eight hours after treating day 1 adult wild‐type or mutant animals with water or 100 mm paraquat, RNA was harvested. mRNA levels of *sod* genes were evaluated by qRT‐PCR and plotted as fold induction (PQ/water) (*y*‐axis) for each *sod* gene. (C) Fluoxetine represses expression of the P*gst‐4::* GFP. Images show GFP fluorescence of P*gst‐4::* GFP reporter animals treated with increasing concentrations of Fluoxetine alone, or in combination with 50 μm Mianserin, on day 1 of adulthood. Images were captured 24 h after treatment. (D) Quantification of (C) Fluoxetine‐induced repression of *Pgst‐4::* GFP. Fluorescent intensity [A.U.] (*y*‐axis) is plotted as a function of Fluoxetine concentration [μm] (*x*‐axis). All error bars show SEM of 3–6 independent experiments. For detailed statistics, see Table S6 (Supporting information). *P<0.05, **P<0.01, ***P<0.001, n.s., not significant P>0.05.

We next asked whether activation of the oxidative stress response strictly depends on synaptic transmission or whether there are nervous system‐dependent and nervous system‐independent activation mechanisms (Staab *et al*., 2014). To test whether ROS activates the oxidative stress response independently of synaptic transmission, we measured P*gst‐4*::GFP expression in wild‐type animals and in animals with decreased synaptic transmission after an 8‐h paraquat exposure (Fig. 4A). Paraquat activated P*gst‐4*::GFP expression in wild‐type, *unc‐26(e205),* and *snt‐1(md290)* mutants (Fig. 4A, Table S6), suggesting a synapse‐independent activation of the oxidative stress response. To corroborate this result, we also measured the activation of *sod* genes by qRT‐PCR. As with the P*gst‐4*::GFP reporter (Fig. 4A), activation of the *sod* genes by paraquat did not depend on synaptic transmission (Fig. 4B). To distinguish the two activation mechanisms, we henceforth will refer to them as ROX, for the ROS‐activated response to oxidative stress (Fig. 4), and NEUROX for the neuronally regulated response to oxidative stress (Fig. 3).

To test the ability of NEUROX to repress the oxidative stress response, we reduced synaptic transmission (Fig. 1B) by treating P*gst‐4*::GFP reporter animals with increasing concentrations of Fluoxetine (Fig. 4C). This resulted in a dose‐dependent reduction of P*gst‐4*::GFP. However, due to the low basal levels in wild‐type background, only a mild reduction was observed with Fluoxetine (Fig. 4C,D). We therefore combined increasing concentrations of Fluoxetine along with induction of P*gst‐4*::GFP reporter with 50 μm Mianserin. Fluoxetine effectively repressed P*gst‐4*::GFP induction by Mianserin (Fig. 4C,D) and similarly repressed the elevated P*gst‐4*::GFP expression seen in *dgk‐1(ok1462)* mutants. These experiments confirm that, depending on the synaptic transmission activity, NEUROX can activate or repress the oxidative stress response in peripheral tissues (see Discussion).

Taken together, these results show that ROS generators such as paraquat and antidepressants modulate the oxidative stress response via two distinct mechanisms. Paraquat activates the oxidative stress response by a synapse‐independent cell‐autonomous mechanism (ROX) (Fig. 4), while antidepressants activate or repress the oxidative stress response by a mechanism dependent on synaptic transmission (NEUROX) (Figs 1, 2, 3).

### Mianserin‐induced extension of lifespan depends on NEUROX

**Figure 5:**
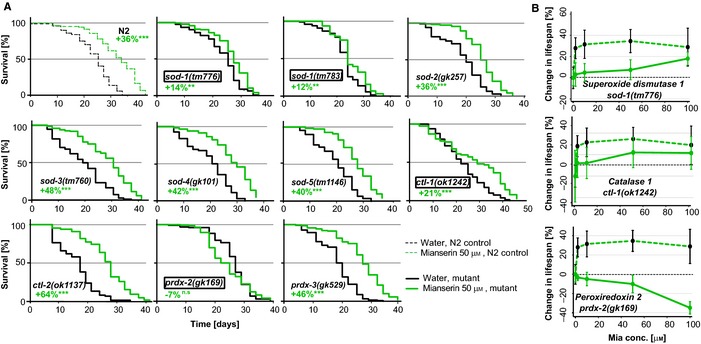
Lifespan extension and protection from oxidative stress by Mianserin require similar genes. (A) Mianserin partially requires *sod‐1 and ctl‐1* and completely requires *prdx‐2,* but does not require *sod‐2, sod‐3, sod‐4*,* sod‐5, ctl‐2, ctl‐3,* and *prdx‐6* for lifespan extension. N2 animals (dotted lines) and animals carrying deletion mutations in redox genes (solid lines), namely *sod‐1(tm776)*,* sod‐1(tm783)*,* sod‐2(gk257)*,* sod‐3(tm760)*,* sod‐4(gk101)*,* sod‐5(tm1146)*,* ctl‐1(ok1242), ctl‐2(ok1137)*,* ctl‐3(ok2042), prdx‐2 (gk169),* and *prdx‐3(gk529),* were treated with water (black) or 50 μm Mianserin (green) on day 1, and their lifespans were assessed. Graphs show fraction of animals alive [%] (*y*‐axis) as a function of time [days] (*x*‐axis). See Table S9 for statistics of all lifespan trials. (B) Lifespan extension by Mianserin is reduced or abolished in mutants with an impaired oxidative stress response. Graph shows mean increase in lifespan [%] (*y*‐axis) as a function of Mianserin concentration [μm] (*x*‐axis). Dotted green lines represent Mianserin‐treated wild‐type (N2) animals, and solid green lines represent animals carrying mutations in oxidative stress response genes required for NEUROX. Genotype for each strain is indicated in each graph. All error bars show standard deviation for one experiment. The response of each mutant was tested in at least one additional experiment. For detailed statistics, see Table S7 (Supporting information).

The finding that prompted us to embark on this study was the observation that compounds targeting biogenic amines, such as the antidepressant Mianserin, increased resistance to oxidative stress as well as lifespan (Petrascheck *et al*., 2007; Ye *et al*., 2014). This made us ask whether, and how, the lifespan‐extending effect of Mianserin is mechanistically related to the neuronal activation of the oxidative stress response NEUROX (Fig. 5).

To determine the relationship between lifespan and oxidative stress resistance, we treated mutants defective in NEUROX with Mianserin and conducted lifespan experiments (Fig. 5A). Mianserin showed reduced or no effect on lifespan for all three antioxidant mutants required for NEUROX. In *sod‐1(tm776)* and *ctl‐1(ok1242)* mutants, the lifespan‐extending effect of Mianserin was reduced, while in *prdx‐2(gk169)* mutants, the effect was abolished completely (Fig. 5A, boxes, Table S7), which was further confirmed using dose–response studies with Mianserin for lifespan extension in these three mutants (Fig. 5B). In contrast, Mianserin fully extended lifespan in all superoxide dismutase mutants not required for NEUROX, namely *sod‐2(gk257), sod‐3(tm760), sod‐4(gk101)*,* sod‐5(tm1146), ctl‐1(ok1137), and prdx‐3(gk529)* (Fig. 5A, Table S7). Thus, only oxidative stress response genes that are required for NEUROX, namely *sod‐1*,* prdx‐2,* and *ctl‐1* (Fig. 2), were required by Mianserin to increase lifespan (Fig. 5A,B), while oxidative stress response genes that were specific for ROX, namely *sod‐4* and *sod‐5* (Figs 2B and 4B), were not (Fig. 5, Table S7).

## Discussion

**Figure 6:**
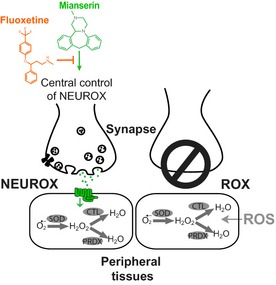
Neuronal versus cell‐autonomous activation of the oxidative stress response. Left synapse: The neuronal regulation of the oxidative stress response (NEUROX) directly or indirectly regulates transcription of oxidative stress response genes via neuronal synaptic transmission. NEUROX is differentially modulated by different antidepressants, with Mianserin activating and Fluoxetine inhibiting the oxidative stress response. Right synapse: In animals with impaired synaptic transmission, the neuronal control of the oxidative stress response is lost and reactive oxygen species activate the oxidative stress response cell autonomously (ROX).

In this study, we used a chemical genetics approach to study aging and stress in *C. elegans*. We investigated the ability of different classes of antidepressants to modulate the response to oxidative stress by combining antidepressants with mutations in classical stress response genes or synaptic transmission genes. The results unveiled the existence of the neuronal control of the oxidative stress response (NEUROX) (Fig. 6). NEUROX is activated by the *atypical* antidepressant Mianserin and is inhibited by the *typical* antidepressant Fluoxetine in a non‐cell‐autonomous manner (Figs 1, 2, 3, 4). NEUROX differs from the ROS‐activated oxidative stress response ROX, in that, it requires synaptic transmission (Figs 3, 4 and 6). The lifespan‐extending effect of Mianserin (Petrascheck *et al*., 2007; Ye *et al*., 2014) (Fig. 5) functionally overlaps with NEUROX, as all the stress response genes required for NEUROX (Fig. 2) are also required for the lifespan‐extending effect (Fig. 5), while genes dispensable for stress protection are dispensable for lifespan extension.

### How does the Mianserin‐induced oxidative stress resistance relate to longevity?

In this study, we set out to investigate the mechanistic basis by which antidepressants such as Mianserin increase resistance to oxidative stress, and how this activation relates to lifespan. We envisioned two different hypotheses to describe the relationship between oxidative stress and lifespan: (i) Mianserin increases lifespan and stress resistance by independent mechanisms; (ii) Mianserin increases resistance to oxidative stress and lifespan by an overlapping mechanism.

Our data are consistent with the latter hypothesis (ii), as Mianserin‐induced lifespan extension and stress resistance require a common set of genes. The mutant strains *sod‐1(tm776), ctl‐1(ok1242)*, and *prdx‐2(gk169)* show an impaired oxidative stress response and are required for the full lifespan extension by Mianserin. What is remarkable is that we have uncovered two independent activation mechanisms for the oxidative stress response, one of which (NEUROX) is required for lifespan extension by Mianserin and the other (ROX) is not. In accordance with the idea of correlating lifespan and functional improvement, it is also important to highlight that at least pharmacologically, longevity and oxidative stress resistance are only correlated with compounds targeting biogenic amines, and not with compounds of other pharmacological classes (e.g., kinase inhibitors) (Ye *et al*., 2014; Bansal *et al*., 2015).

### Cell‐autonomous versus non‐cell‐autonomous regulation of the oxidative stress response by ROX and NEUROX

The results presented in this study provide multiple lines of evidence for the existence of two very distinct modes of activation of the oxidative stress response: ROX, which stands for ROS‐activated response to oxidative stress, and NEUROX, which stands for neuronally regulated response to oxidative stress. The main difference is that NEUROX is non‐cell‐autonomous and regulated by synaptic transmission (Fig. 3), while ROX is likely to be cell‐autonomous (Fig. 4). The two mechanisms are genetically distinct, as established by the following results: (i) Enhancing synaptic transmission with Mianserin increases the expression of oxidative stress response genes (Figs 1, 2, 3); (ii) decreasing synaptic transmission with Fluoxetine decreases the oxidative stress response (Figs 1 and 4); (iii) the Mianserin‐induced protection from oxidative stress is abolished in synaptic mutants such as *unc‐26(e205)*,* snt‐1(md290), snb‐1(md247), unc‐18(e81), unc‐2(e55), unc‐10(e102), and unc‐11(e47),* and in the chemosensation mutant *che‐12(e1812)* (Fig. 3); and (iv) paraquat activates the oxidative stress response independently of synaptic transmission (Fig. 4). The ability of NEUROX to activate as well as inhibit the oxidative stress response (Figs 1, 3 and 4) may also explain how some synaptic mutants show an increased resistance to oxidative stress while others show a decreased resistance (Fig. 3). Another possibility is that these differences in stress resistance arise from pleiotropic effects. Whatever the reasons, none of the synaptic mutants were able to increase resistance in response to Mianserin.

Why would the organism need two different mechanisms? ROX is activated by the actual presence of ROS to limit oxidative stress‐induced damage. In contrast, NEUROX is likely activated in anticipation of oxidative stress, yet to come. NEUROX thereby generates capacity to deal with impending stress and, hence, dramatically increases the animal's resistance to oxidative stress. Capacity to deal with oxidative stress could be generated by a constitutive activation of the oxidative stress response, or by a homeostatic change that primes the oxidative stress response to be triggered by lower ROS concentrations. At this point, we consider priming for enhanced activation as the more likely model because of two reasons. First, a 2‐h Mianserin treatment increases synaptic transmission (Fig. 1B), but is not sufficient to induce the *Pgst‐4*::GFP reporter, while a 2‐h paraquat treatment is (Fig. S2). This delay in activation shows that Mianserin does not directly trigger the oxidative stress response and therefore induces a homeostatic change that takes time. Second, and most importantly, in animals with a prolonged increase in synaptic transmission (*dgk‐1(ok1462)*, Fig. 3B,C), the dose–response profile of *Pgst‐4::*GFP activation is dramatically shifted to the left and the response amplitude is increased. Prolonged increase in synaptic transmission (*dgk‐1(ok1462),* Fig. 3B,C) therefore results in a homeostatic change that tunes the oxidative stress response to be triggered at lower concentrations than observed in wild‐type animals (wt, Fig. 3B,C). Thus, it is likely that NEUROX evolved as a preemptive mechanism that tunes the activation threshold necessary to trigger the oxidative stress response in response to adverse conditions, detected by sensory perception. The finding that chemosensation is required (*che‐12(e1812),* Fig. 3F,G) for NEUROX supports this model. It will be interesting to see whether hormetic lifespan extensions trigger NEUROX, rather than ROX, thus extending lifespan, as the nervous system primes the stress response to be triggered at lower concentrations. If true, this model would predict that hormetic mechanisms would depend on synaptic transmission as well.

The ability of antidepressants to modulate NEUROX raises the possibility that NEUROX is part of a larger survival mechanism. The fight‐or‐flight response, for example, ensures survival by changing behavior in response to perceived dangers (Graeff, 1994). Interestingly, the fight‐or‐flight response is associated with anxiety and stress behaviors (Graeff, 1994), which are the very behaviors suppressed by anxiolytic action of antidepressants such as Mianserin and Mirtazapine, prescribed to patients. Therefore, it is conceivable that such behavioral responses also include physiological changes that modulate the oxidative stress response via NEUROX as a protective mechanism.

The ideas proposed above raise the question of whether antidepressants protect from oxidative stress in mammals. In support of these ideas, depressogenic paradigms causing ‘psychological’ stress in rodents (e.g., restraint stress) have been shown to generate oxidative damage leading to the accumulation of malondialdehydes (MDA) or 4‐hydroxy‐2‐nonenals (4‐HNE). Consistent with our findings that Mianserin protects from oxidative stress in *C. elegans*, antidepressant treatment in rodents suppresses the accumulation of oxidative stress markers MDA and 4‐HNE generated by restraint stress in rodents (Abdel‐Wahab & Salama, 2011; Maes *et al*., 2011; Spiers *et al*., 2013). Similarly, patients suffering from major depression show increased oxidative damage and reduced antioxidant capacity in the blood (Scapagnini *et al*., 2012).

Conversely, antioxidants have recently been shown to have antidepressant‐like effects. Multiple antioxidants, such as N‐acetyl‐cysteine (NAC), glutathione, and tocopherol, have antidepressant‐like effects in rodents in the tail suspension test. Furthermore, in patients, NAC was found to have antidepressant‐like effects in randomized clinical trials (Maes *et al*., 2011). Interestingly, dietary restriction, a well‐established method to extend lifespan that also reduces oxidative damage, was found to have antidepressant effects in mice (Lutter *et al*., 2008). This links lifespan, stress resistance, and antidepressant‐like effects (Lutter *et al*., 2008), in a manner similar to what we have shown for Mianserin in our studies.

To be prudent, it has to be pointed out that oxidative stress is associated with nearly every disease condition and any causal role in antidepressant action has yet to be determined. However, more directly related to our findings are two recent studies demonstrating that Mirtazapine treatment protects kidneys from oxidative damage caused by ischemia–reperfusion injury and neurons from oxidative damage caused by cisplatin, in rats (Tok *et al*., 2012; Gulec *et al*., 2013).

Our finding that antidepressants modulate the oxidative stress response via NEUROX in *C. elegans* raises the exciting possibility that NEUROX may be part of the mechanistic basis of some clinically relevant antidepressants, and that some antidepressants induce physiological changes that may have therapeutic uses outside of depression.

## Experimental procedures

### Chemicals

Solvents used to prepare stock solutions are as follows: Mianserin, Fluoxetine, and paraquat were dissolved in water; Mirtazapine and serotonin antagonists were dissolved in DMSO; FUDR was dissolved in S‐complete; and Aldicarb was dissolved in ethanol. For supplier, catalog, and CAS numbers for each chemical, see extended methods in the supplementary material.

### Strains

Detailed descriptions of all strains used in this study are available in Appendix S1. Mutant strains were backcrossed at least four times with the N2 Bristol strain. All strains were maintained as described (Brenner 1974).

### Lifespan assay

Lifespan assays were conducted in 96‐well plates as described in Solis & Petrascheck (2011) and Rangaraju *et al*. (2015). When used, DMSO was kept to a final concentration of 0.33% v/v. Animals were scored three times a week, until 95% of animals were dead in all the tested conditions. Statistical analysis was performed using the Mantel–Haenszel version of the log‐rank test.

### Stress resistance assays

Resistance to oxidative stress was determined by measuring the survival of antidepressant‐treated and untreated worms after a 24‐h exposure to the ROS generator paraquat (methyl viologen). Experimental worm cultures were set up as described in lifespan assays. Solvent (water or DMSO) or antidepressants were added on day 1 of adulthood, unless otherwise indicated. Paraquat was added at increasing concentrations on day 5 of adulthood, unless otherwise indicated. For all experiments, the survival of worms was assessed 24 h after paraquat addition and expressed as the percentage of live versus total animals. For the serotonin antagonists experiment, heat map and hierarchical clustering analysis (dendrograms) were generated with R using the heatmap.2 function of the gplots package. Dissimilarity between values and hierarchical clustering were calculated using the Euclidean distance measure and complete linkage method.

### *In vitro* radical‐scavenging assays

Free radical‐scavenging activity of antidepressants was measured in a cell‐free assay using DPPH as a free radical and Trolox as a positive control scavenger. The DPPH radical absorbs light at 520 nm, while its reduced form does not, allowing to monitor scavenging activity (Pisoschi *et al*., 2009). Mianserin, Mirtazapine, and Trolox stock solutions (10x) were prepared in ethanol (see Appendix S1 for catalog numbers and chemical information) and tested at increasing concentrations for their ability to scavenge DPPH (90 μm) using ethanol as a negative control. Averages between triplicates were calculated, subtracted from the blank, and normalized to the absorbance of DPPH with ethanol alone. All values were plotted as DPPH [%] against dual *x*‐axis of log [10] concentration of drug and log [10] ratio of small molecule to DPPH.

### Real‐time quantitative PCR (qRT‐PCR) and data analysis

All qRT‐PCR experiments were conducted according to the MIQE guidelines (Bustin *et al*., 2009), except that samples were not tested in a bio‐analyzer. RNA was extracted on day 5 of adulthood, as described above, followed by DNase treatment and reverse transcription at 42 °C for 30 min. Five microliters of quantitative PCRs was set up in 384‐well plates using the following thermocycler settings: [95 °C, 3 min]; 40 × [95 °C 10 s, 60 °C 30 s]; and melting curve: 95 °C 5 s, 60–95 °C at 0.5 °C increment, 10 s (see Appendix S1 for catalog numbers and reagent information). Gene expression was normalized to three reference genes, *rcq‐5*,* crn‐3,* and *rpl‐6,* using the bio‐rad cfx manager software (BIO‐RAD, Hercules, CA, USA). Statistical significance was determined using *t*‐test.

### Aldicarb assay

Synaptic transmission in response to the antidepressants Mianserin and Fluoxetine was measured by determining onset of paralysis by the acetylcholine esterase inhibitor aldicarb. For each condition, 60–80 L4‐stage animals were treated with water, Mianserin (50 μm), or Fluoxetine (100 μm) followed by aldicarb addition (4 mm final conc.) 2 h later. Paralysis of animals was scored visually, every 15 min. Results were graphed as the paralyzed fraction of animals [%], as a function of time. The mean paralysis [%] from six independent experiments was calculated, and statistical significance was determined using Student's *t*‐test.

### Immunoblotting

Immunoblotting was conducted to verify changes in GFP expression. Worms were cultured in 96‐well plates, as described in lifespan analysis, treated with Mianserin (50 μm) or water on day 1 of adulthood, and harvested on day 5. Harvested worms (~500 worms) were washed and lysed in a Precellys lysing system. Protein concentrations were determined; equal amounts of protein (20 μg per lane) were separated by SDS‐PAGE and transferred to nitrocellulose membranes. Membranes were blocked and incubated overnight in rabbit anti‐GFP primary antibody. After washing, goat anti‐rabbit IRdye 800CW (LI‐COR) was added for 1 h. After washing, bound antibodies were visualized and imaged using LI‐COR IR dye detection system. For loading control, mouse anti‐actin primary antibody was used, followed by goat anti‐mouse IRdye 800CW (LI‐COR) secondary antibody. The GFP and actin band intensities were quantified using LI‐COR Odyssey quantification software. Statistical significance was determined using *t*‐test from three to five independent experiments.

### Fluorescent microscopy, imaging, and quantification

Reporter strains were cultured in 96‐well plates (Solis & Petrascheck, 2011; Rangaraju *et al*., 2015) and exposed to compounds on day 1 of adulthood. Concentrations varied dependent on compound: Mianserin was used at 0, 2, 10, 50, 100, or 200 μm; Fluoxetine was used at 0, 50, 100, 250, or 500 μm; and paraquat was used at 100 mm. Fluorescent images were taken 24 h after Mianserin or Fluoxetine treatment and 8 h after paraquat treatment. For imaging, animals were washed several times to remove eggs and bacteria using paralysis solution (0.3 mg mL^−1^ levamisole, 0.005% Triton X‐100 in M9), pooled (8–16 wells per condition ~100–150 worms), and transferred into a well of a black, clear‐bottom imaging plate (Shore *et al*., 2012). Bright‐field and fluorescent images were captured by a Molecular Devices ImageXpress (Molecular Devices, Sunnyvale, CA, USA) platform. Fluorescence for each worm was quantified by the cell profiler software (www.cellprofiler.org) using the bright‐field image to create a mask for each worm, used to measure the mean fluorescence per area after median background subtraction. Statistical significance was determined using *t*‐test.

## Author contributions

M.P. and S.R. conceived, designed, and planned the studies. A.B.N. contributed intellectually to the study. S.R. outcrossed the strains and generated the reporter strains. S.R. performed the qRT‐PCR, immunoblotting, and lifespan experiments. S.R., G.M.S., and M.P. performed the high‐content imaging, aldicarb assays, and qRT‐PCR sample collection. S.R., G.M.S., R.G.A., C.D.B., and M.P. performed the stress resistance assays. S.I.A. performed the DPPH assays. R.K. was involved in outcrossing strains and lifespan experiments. S.R. and M.P. performed data analyses. M.P. and S.R. interpreted the results, prepared the figures and tables, and wrote the manuscript.

## Funding

No funding information provided.

## Conflict of interest

The authors declare no conflict of interests.

## Supporting information


**Fig. S1** Stress protection by Mianserin is not due to inability of animals to intake paraquat. Related to Fig. 1.
**Fig. S2** Mianserin does not induce P*gst‐4*::GFP at 2 h. Related to Fig. 2.
**Fig. S3** Mianserin does not require *daf‐16* but involves *skn‐1* for stress protection. Related to Fig. 2.
**Table S1** Paralysis data for aldicarb assays. Related to Fig. 1B.
**Table S2** Survival data for paraquat stress resistance assays. Related to Fig. 1C.
**Table S3** Survival data for paraquat stress resistance assays. Related to Figs 1D and 2B.
**Table S4** Survival data for paraquat stress resistance assays. Related to Fig. 3F.
**Table S5** Summary of oxidative stress protection by serotonin antagonists. Related to Fig. 3H.
**Table S6** Summary of fluorescence intensity quantification for P*gst‐4*::GFP reporter after paraquat treatment. Related to Fig. 4A.
**Table S7** Summary of all lifespan data for Mianserin. Related to Fig. 5.
**Appendix S1** Extended experimental procedures
