# Supplementary material for: Atypical antidepressants extend lifespan of Caenorhabditis elegans by activation of a non‐cell‐autonomous stress response
Source: Aging Cell. 2015 Aug 8;14(6):971–81. doi: 10.1111/acel.12379 (PMC4693466; doi:10.1111/acel.12379)
Supplement: Supplementary file 1 — Fig. S1 Stress protection by Mianserin is not due to inability of animals to intake paraquat. Related to Fig. 1. Fig. S2 Mianserin does not induce Pgst‐4::GFP at 2 h. Related to Fig. 2. Fig. S3 Mianserin does not require daf‐16 but involves skn‐1 for stress protection. Related to Fig. 2. Table S1 Paralysis data for aldicarb assays. Related to Fig. 1B. Table S2 Survival data for paraquat stress resistance assays. Related to Fig. 1C. Table S3 Survival data for paraquat stress resistance assays. Related to Figs 1D and 2B. Table S4 Survival data for paraquat stress resistance assays. Related to Fig. 3F. Table S5 Summary of oxidative stress protection by serotonin antagonists. Related to Fig. 3H. Table S6 Summary of fluorescence intensity quantification for Pgst‐4::GFP reporter after paraquat treatment. Related to Fig. 4A. Table S7 Summary of all lifespan data for Mianserin. Related to Fig. 5. Appendix S1 Extended experimental procedures [file ACEL-14-0971-s001.docx]

**Supplemental Information**

**Supplemental Figure Legends:**

**
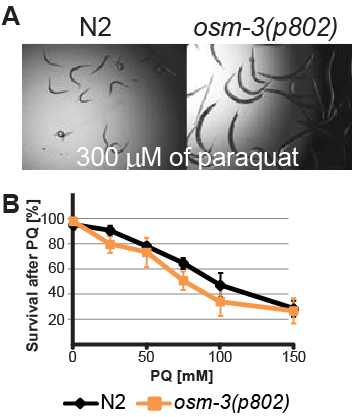
**

**Figure S1: Stress protection by Mianserin is not due to inability of animals to intake paraquat. Related to Figure 1.**

A) Paraquat does not hinder development in *osm-3(p802)* mutants. Bright field images of synchronized L1 worms that were seeded into 96-well plates, treated with 300 µM paraquat and incubated at 20°C for 72 h.

B) Wild type (N2) or *osm-3(p802)* animals were treated on day 5 of adulthood with increasing concentrations of paraquat (PQ). Survival of animals after 24 h paraquat (PQ) [%] (Y-axis) as a function of PQ concentration [mM] (X-axis) is shown. All error bars show S.E.M of 3 independent experiments.


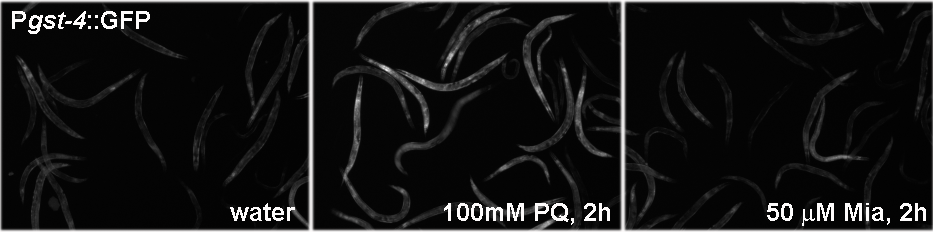


**Figure S2: Mianserin does not induce P*gst-4*::GFP at 2 h. Related to Figure 2.**

Mianserin does not induce expression of the P*gst-4*::GFP reporter at a shorter treatment time. Images show GFP fluorescence of day 1 adults treated with water, paraquat (100mM) for 2h or Mianserin (50 µM) for 2h. Images were captured after the 2h treatment using identical exposure settings.

**
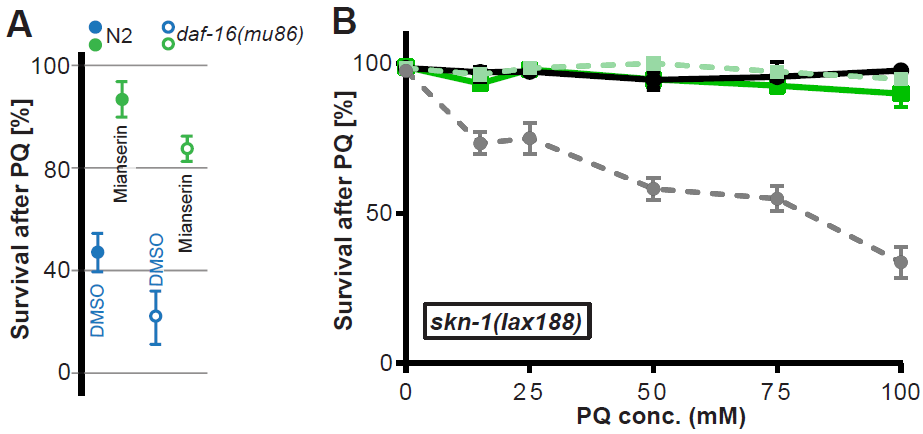
**

**Figure S3: Mianserin does not require *daf-16* but involves *skn-1* for stress protection. Related to Figure 2.**

A) DAF-16 transcription factor is not required for Mianserin-induced stress resistance. DMSO- (blue) and Mianserin-treated (green) of N2 (solid circle) and *daf-16(mu86*) (hollow circle) are indicated. Graph shows the percent survival of animals [%] (Y-axis) after 24 h paraquat (75 mM) exposure. Error bars show S.E.M of 3 independent experiments.

B) Mianserin-induced protection from oxidative stress requires constitutive skn-1 activity. Wild-type (dotted lines) or skn-1(lax188) (bold lines) day 1 adults were treated with water or 50 µM Mianserin, followed by increasing concentrations of paraquat on day 5. Survival of animals was determined 24 h later and plotted in [%] (Y-axis) as a function of paraquat concentration [mM] (X-axis).

**Supplemental Tables:**

**Table S1: Paralysis data for aldicarb assays. Related to Figure 1B.**

| **Treatment** | **Time [min]** | **Paralysis [%] Expt.1** | **Paralysis [%] Expt.2** | **Paralysis [%] Expt.3** | **Paralysis [%] Expt.4** | **Paralysis [%] Expt.5** | **Paralysis [%] Expt.6** | **Mean,**  **Paralysis [%]** | **S.D.,**  **Paralysis [%]** | **P-value** | **No. of wells** | **Total no. of animals** |
| --- | --- | --- | --- | --- | --- | --- | --- | --- | --- | --- | --- | --- |
| water | 0 | 0.0 | 0.0 | 0.0 | 0.0 | 0.0 | 0.0 | **0.0** | 0.0 |  | 48 | 366 |
|  | 15 | 0.0 | 0.0 | 0.0 | 0.0 | 0.0 | 0.0 | **0.0** | 0.0 |  |  |  |
|  | 30 | 4.9 | 9.5 | 0.0 | 29.4 | 1.1 | 12.8 | **9.6** | 10.9 |  |  |  |
|  | 45 | 54.4 | 67.0 | 50.5 | 85.0 | 58.9 | 54.5 | **61.7** | 12.7 |  |  |  |
|  | 60 | 86.5 | 81.6 | 84.0 | 91.9 | 70.1 | 75.5 | **81.6** | 7.8 |  |  |  |
|  | 75 | 89.3 | 90.1 | 70.0 | 93.5 | 68.2 | 92.5 | **83.9** | 11.6 |  |  |  |
| Mianserin 50 µM | 0 | 0.0 | 0.0 | 0.0 | 0.0 | 0.0 | 0.0 | **0.0** | 0.0 | **N/A** | 48 | 358 |
|  | 15 | 28.9 | 0.0 | 57.7 | 0.0 | 27.8 | 28.9 | **23.9** | 21.7 | **4.29E-02** |  |  |
|  | 30 | 71.1 | 70.2 | 75.5 | 70.2 | 71.5 | 71.1 | **71.6** | 2.0 | **2.27E-05** |  |  |
|  | 45 | 85.6 | 75.1 | 98.2 | 84.1 | 81.3 | 85.6 | **85.0** | 7.6 | **4.72E-03** |  |  |
|  | 60 | 96.5 | 94.2 | 98.2 | 96.3 | 95.1 | 96.5 | **96.1** | 1.4 | **5.61E-03** |  |  |
|  | 75 | 98.2 | 88.0 | 100.0 | 89.7 | 100.0 | 98.2 | **95.6** | 5.4 | **0.06** |  |  |
| Fluoxetine 100 µM | 0 | 0.0 | 0.0 | 0.0 | 0.0 | 0.0 | 0.0 | **0.0** | 0.0 | **N/A** | 48 | 393 |
|  | 15 | 0.0 | 0.0 | 0.0 | 0.0 | 0.0 | 0.0 | **0.0** | 0.0 | **N/A** |  |  |
|  | 30 | 1.3 | 0.0 | 0.0 | 4.3 | 0.0 | 4.6 | **1.7** | 2.2 | **0.14** |  |  |
|  | 45 | 32.9 | 12.4 | 8.2 | 39.6 | 5.7 | 18.9 | **19.6** | 13.8 | **2.70E-04** |  |  |
|  | 60 | 45.5 | 47.5 | 25.1 | 59.4 | 19.7 | 42.5 | **40.0** | 14.9 | **3.69E-04** |  |  |
|  | 75 | 50.2 | 74.0 | 49.2 | 90.0 | 51.1 | 63.5 | **63.0** | 16.4 | **3.11E-02** |  |  |

Summary of all aldicarb paralysis assays performed in Figure 1B. The treatments, water, Mianserin 50 µM, or Fluoxetine 100 µM were added on L4 stage for 2 h. Aldicarb (4 mM) was added after 2 h and paralysis [%] was assessed in 15 min intervals. Mean and standard deviation (S.D.) of paralysis [%] were calculated from 6 independent experiments (expts.). P-values were calculated between water and small molecule treatment samples, for each time point, using unpaired *t-test*. The total number of wells and animals from which data were collected are indicated.

**Table S2: Survival data for paraquat stress resistance assays. Related to Figure 1C.**

| **Strain** | **Treatment** | **Conc. [µM]** | **PQ conc. [mM]** | **Survival after PQ [%]**  **(expt. 1)** | **Survival after PQ [%]**  **(expt. 2)** | **Survival after PQ [%]**  **(expt. 3)** | **Survival after PQ [%]**  **(expt. 4)** | **Mean,**  **Survival after PQ [%]** | **S.D.,**  **Survival after PQ [%]** | **P-value** | **No. of wells** | **Total no. of animals** |
| --- | --- | --- | --- | --- | --- | --- | --- | --- | --- | --- | --- | --- |
| N2 | water | 0 | 100 | 52.7 | 44.7 | 49.3 |  | **48.9** | 4.0 |  | 103 | 1087 |
| N2 | Mianserin | 2 | 100 | 76.3 | 79.9 | 80.6 |  | **78.9** | 2.3 | **1.17E-03** | 34 | 353 |
| N2 | Mianserin | 10 | 100 | 85.4 | 95.5 | 85.2 |  | **88.7** | 5.9 | **1.12E-03** | 34 | 358 |
| N2 | Mianserin | 50 | 100 | 90.0 | 78.3 | 91.7 |  | **86.7** | 7.3 | **3.8E-03** | 34 | 335 |
| N2 | Mianserin | 100 | 100 | 85.9 | 88.8 | 93.7 |  | **89.5** | 4.0 | **2.42E-04** | 31 | 299 |
| N2 | DMSO | 0 | 100 | 49.4 | 44.9 | 49.3 |  | **47.9** | 2.6 |  | 92 | 951 |
| N2 | Mirtazapine | 2 | 100 | 56.5 | 47.6 | 41.1 |  | **48.4** | 7.7 | **0.92** | 25 | 278 |
| N2 | Mirtazapine | 10 | 100 | 74.7 | 78.8 | 75.0 |  | **76.2** | 2.3 | **1.56E-04** | 28 | 276 |
| N2 | Mirtazapine | 50 | 100 | 83.8 | 78.7 | 82.2 |  | **81.6** | 2.6 | **9.21E-05** | 27 | 292 |
| N2 | Mirtazapine | 100 | 100 | 82.1 | 83.1 | 74.0 |  | **79.7** | 5.0 | **2.24E-03** | 30 | 328 |
| N2 | water | 0 | 100 | 60.0 | 48.9 | 48.7 | 50.9 | **52.1** | 5.4 |  | 66 | 954 |
| N2 | Fluoxetine | 2 | 100 | 54.9 | 57.5 | 52.7 |  | **55.0** | 2.4 | **0.58** | 20 | 321 |
| N2 | Fluoxetine | 10 | 100 | 46.8 | 57.1 | 40.4 |  | **48.1** | 8.4 | **0.51** | 31 | 487 |
| N2 | Fluoxetine | 50 | 100 | 41.1 | 47.0 | 38.2 | 42.0 | **42.1** | 3.6 | **0.09** | 38 | 435 |
| N2 | Fluoxetine | 100 | 100 | 29.1 | 41.0 | 24.8 | 45.0 | **35.0** | 9.6 | **3.00E-02** | 40 | 544 |

Summary of all stress resistance assays performed in Figure 1C. The treatments, water, Mianserin, DMSO, Mirtazapine or Fluoxetine, at their indicated concentrations (conc.) were added on day 1 of adulthood. Paraquat (PQ) was added to a final conc. of 100 mM on day 5 and survival [%] was calculated 24 h later. Mean and standard deviation (S.D.) of survival after PQ [%] were calculated from independent experiments (expts.). P-values were calculated between control (water or DMSO) and small molecule treatment samples using unpaired *t-test*. The total number of wells and animals from which data were collected are indicated.

**Table S3: Survival data for paraquat stress resistance assays. Related to Figure 1D and 2B.**

| **Strain** | **Treatment** | **Conc. [µM]** | **PQ conc. [mM]** | **Survival after PQ [%]**  **(expt. 1)** | **Survival after PQ [%]**  **(expt. 2)** | **Survival after PQ [%]**  **(expt. 3)** | **Survival after PQ [%]**  **(expt. 4)** | **Survival after PQ [%]**  **(expt. 5)** | **Survival after PQ [%]**  **(expt. 6)** | **Survival after PQ [%]**  **(expt. 7)** | **Mean,**  **Survival after PQ [%]** | **S.D.,**  **Survival after PQ [%]** | **P-value** | **No. of wells** | **Total no. of animals** |
| --- | --- | --- | --- | --- | --- | --- | --- | --- | --- | --- | --- | --- | --- | --- | --- |
| **N2** | water | 0 | 0 | 100 | 95.6 | 85.5 | 87.4 | 95.0 | 100 | 97.4 | **94.4** | 5.8 |  | 56 | 461 |
|  | water | 0 | 15 | 95.0 | 87.2 | 78.6 | 70.7 | 83.8 | 92.9 | 95.9 | **86.3** | 9.3 |  | 56 | 460 |
|  | water | 0 | 25 | 78.0 | 75.4 | 70.1 | 61.4 | 73.2 | 88.6 | 79.3 | **75.1** | 8.4 |  | 56 | 425 |
|  | water | 0 | 50 | 64.2 | 44.7 | 68.8 | 49.8 | 55.3 | 85.1 | 76.7 | **63.5** | 14.6 |  | 56 | 403 |
|  | water | 0 | 75 | 58.4 | 24.7 | 76.6 | 52.2 | 50.5 | 75.0 | 64.2 | **57.4** | 17.6 |  | 56 | 444 |
|  | water | 0 | 100 | 31.4 | 13.9 | 53.8 | 48.0 | 51.8 | 62.5 | 61.4 | **46.1** | 17.6 |  | 56 | 456 |
|  | Mia | 50 | 0 | 98.6 | 100 | 98.2 | 100 | 100 | 100 | 100 | **99.5** | 0.8 | **0.06** | 56 | 410 |
|  | Mia | 50 | 15 | 100 | 98.0 | 98.9 | 98.9 | 97.9 | 100 | 97.9 | **98.8** | 0.9 | **1.21E-02** | 56 | 432 |
|  | Mia | 50 | 25 | 97.9 | 96.8 | 96.7 | 98.9 | 96.4 | 98.8 | 93.2 | **96.9** | 1.9 | **3.57E-04** | 56 | 435 |
|  | Mia | 50 | 50 | 100 | 70.2 | 96.9 | 97.5 | 78.8 | 97.2 | 97.9 | **91.2** | 11.7 | **2.24E-03** | 56 | 446 |
|  | Mia | 50 | 75 | 93.5 | 59.0 | 96.8 | 91.6 | 76.6 | 96.8 | 96.7 | **87.3** | 14.4 | **4.85E-03** | 56 | 424 |
|  | Mia | 50 | 100 | 56.4 | 39.0 | 85.6 | 88.7 | 78.6 | 97.0 | 86.9 | **76.0** | 20.7 | **1.33E-02** | 56 | 381 |
| ***sod-1***  ***(tm776)*** | water | 0 | 0 | 89.0 | 91.9 | 94.2 |  |  |  |  | **91.7** | 2.6 |  | 24 | 235 |
|  | water | 0 | 15 | 73.3 | 58.6 | 69.4 |  |  |  |  | **67.1** | 7.6 |  | 24 | 209 |
|  | water | 0 | 25 | 47.4 | 10.7 | 55.6 |  |  |  |  | **37.9** | 23.9 |  | 24 | 242 |
|  | water | 0 | 50 | 6.8 | 0.0 | 12.3 |  |  |  |  | **6.4** | 6.2 |  | 24 | 218 |
|  | water | 0 | 75 | 0.0 | 0.0 | 2.5 |  |  |  |  | **0.8** | 1.4 |  | 24 | 207 |
|  | water | 0 | 100 | 0.0 | 0.0 | 1.3 |  |  |  |  | **0.4** | 0.8 |  | 24 | 208 |
|  | Mia | 50 | 0 | 100 | 98.2 | 98.0 |  |  |  |  | **98.7** | 1.1 | **2.86E-02** | 24 | 212 |
|  | Mia | 50 | 15 | 1.6 | 0.9 | 8.7 |  |  |  |  | **3.7** | 4.3 | **8.38E-04** | 24 | 214 |
|  | Mia | 50 | 25 | 0.0 | 0.0 | 1.0 |  |  |  |  | **0.3** | 0.6 | **0.11** | 24 | 209 |
|  | Mia | 50 | 50 | 0.0 | 0.0 | 12.5 |  |  |  |  | **4.2** | 7.2 | **0.71** | 24 | 213 |
|  | Mia | 50 | 75 | 0.0 | 0.0 | 0.0 |  |  |  |  | **0.0** | 0.0 | **0.42** | 24 | 191 |
|  | Mia | 50 | 100 | 0.0 | 0.0 | 0.0 |  |  |  |  | **0.0** | 0.0 | **0.42** | 24 | 171 |
| ***sod-1***  ***(tm783)*** | water | 0 | 0 | 97.9 | 96.9 | 92.1 |  |  |  |  | **95.6** | 3.1 |  | 24 | 152 |
|  | water | 0 | 15 | 81.7 | 77.9 | 85.6 |  |  |  |  | **81.7** | 3.8 |  | 24 | 144 |
|  | water | 0 | 25 | 51.2 | 68.7 | 80.5 |  |  |  |  | **66.8** | 14.7 |  | 24 | 126 |
|  | water | 0 | 50 | 20.6 | 8.5 | 12.1 |  |  |  |  | **13.7** | 6.2 |  | 24 | 159 |
|  | water | 0 | 75 | 0.0 | 0.0 | 5.3 |  |  |  |  | **1.8** | 3.0 |  | 24 | 153 |
|  | water | 0 | 100 | 0.0 | 0.0 | 0.0 |  |  |  |  | **0.0** | 0.0 |  | 24 | 121 |
|  | Mia | 50 | 0 | 100 | 98.4 | 98.8 |  |  |  |  | **99.1** | 0.8 | **0.19** | 24 | 134 |
|  | Mia | 50 | 15 | 22.1 | 2.7 | 23.4 |  |  |  |  | **16.1** | 11.6 | **5.84E-03** | 24 | 153 |
|  | Mia | 50 | 25 | 0.0 | 0.0 | 0.0 |  |  |  |  | **0.0** | 0.0 | **1.59E-02** | 24 | 149 |
|  | Mia | 50 | 50 | 0.0 | 0.0 | 1.4 |  |  |  |  | **0.5** | 0.8 | **0.06** | 24 | 146 |
|  | Mia | 50 | 75 | 0.0 | 0.0 | 0.0 |  |  |  |  | **0.0** | 0.0 | **0.42** | 24 | 145 |
|  | Mia | 50 | 100 | 0.0 | 0.0 | 0.0 |  |  |  |  | **0.0** | 0.0 | **--** | 24 | 107 |
| ***sod-2***  ***(gk257)*** | water | 0 | 0 | 89.6 | 94.2 | 91.6 | 89.8 |  |  |  | **91.3** | 2.1 |  | 32 | 302 |
|  | water | 0 | 15 | 90.0 | 78.7 | 92.4 | 68.4 |  |  |  | **82.4** | 11.1 |  | 32 | 316 |
|  | water | 0 | 25 | 89.9 | 53.5 | 73.7 | 70.0 |  |  |  | **71.8** | 14.9 |  | 32 | 301 |
|  | water | 0 | 50 | 76.4 | 8.2 | 57.2 | 33.6 |  |  |  | **43.8** | 29.5 |  | 32 | 309 |
|  | water | 0 | 75 | 10.0 | 0.0 | 15.0 | 10.9 |  |  |  | **9.0** | 6.4 |  | 32 | 289 |
|  | water | 0 | 100 | 0.0 | 0.0 | 3.1 | 10.8 |  |  |  | **3.5** | 5.1 |  | 32 | 274 |
|  | Mia | 50 | 0 | 98.9 | 100 | 97.6 | 99.0 |  |  |  | **98.9** | 1.0 | **2.58E-03** | 32 | 299 |
|  | Mia | 50 | 15 | 98.5 | 96.5 | 99.2 | 94.1 |  |  |  | **97.1** | 2.3 | **7.34E-02** | 32 | 303 |
|  | Mia | 50 | 25 | 98.6 | 89.7 | 98.8 | 91.7 |  |  |  | **94.7** | 4.7 | **4.93E-02** | 32 | 297 |
|  | Mia | 50 | 50 | 96.0 | 24.0 | 93.4 | 77.5 |  |  |  | **72.7** | 33.5 | **0.24** | 32 | 301 |
|  | Mia | 50 | 75 | 34.1 | 2.6 | 32.8 | 52.9 |  |  |  | **30.6** | 20.8 | **0.13** | 32 | 284 |
|  | Mia | 50 | 100 | 0.0 | 0.0 | 1.6 | 57.8 |  |  |  | **14.8** | 28.6 | **0.49** | 32 | 233 |
| ***sod-3***  ***(tm760)*** | water | 0 | 0 | 97.4 | 90.8 | 79.4 |  |  |  |  | **89.2** | 9.1 |  | 24 | 228 |
|  | water | 0 | 15 | 81.7 | 73.0 | 62.5 |  |  |  |  | **72.4** | 9.6 |  | 24 | 246 |
|  | water | 0 | 25 | 72.9 | 61.7 | 67.3 |  |  |  |  | **67.3** | 5.6 |  | 24 | 237 |
|  | water | 0 | 50 | 45.6 | 40.2 | 41.4 |  |  |  |  | **42.4** | 2.8 |  | 24 | 238 |
|  | water | 0 | 75 | 18.0 | 15.0 | 46.7 |  |  |  |  | **26.6** | 17.5 |  | 24 | 201 |
|  | water | 0 | 100 | 1.1 | 8.1 | 29.3 |  |  |  |  | **12.8** | 14.6 |  | 24 | 193 |
|  | Mia | 50 | 0 | 98.1 | 98.4 | 93.0 |  |  |  |  | **96.5** | 3.0 | **0.30** | 24 | 232 |
|  | Mia | 50 | 15 | 94.0 | 93.9 | 96.4 |  |  |  |  | **94.8** | 1.4 | **0.05** | 24 | 228 |
|  | Mia | 50 | 25 | 91.3 | 91.9 | 98.8 |  |  |  |  | **94.0** | 4.2 | **3.51E-03** | 24 | 232 |
|  | Mia | 50 | 50 | 86.0 | 74.0 | 88.5 |  |  |  |  | **82.8** | 7.7 | **6.36E-03** | 24 | 175 |
|  | Mia | 50 | 75 | 66.5 | 41.9 | 90.7 |  |  |  |  | **66.4** | 24.4 | **0.09** | 23 | 222 |
|  | Mia | 50 | 100 | 16.4 | 23.0 | 91.8 |  |  |  |  | **43.7** | 41.8 | **0.33** | 24 | 179 |
| ***sod-4***  ***(gk101)*** | water | 0 | 0 | 96.8 | 87.3 | 85.7 |  |  |  |  | **89.9** | 6.0 |  | 24 | 168 |
|  | water | 0 | 15 | 80.3 | 74.6 | 56.6 |  |  |  |  | **70.5** | 12.4 |  | 24 | 162 |
|  | water | 0 | 25 | 73.3 | 57.6 | 65.0 |  |  |  |  | **65.3** | 7.8 |  | 24 | 169 |
|  | water | 0 | 50 | 62.5 | 36.6 | 44.4 |  |  |  |  | **47.8** | 13.3 |  | 24 | 175 |
|  | water | 0 | 75 | 30.8 | 11.9 | 51.1 |  |  |  |  | **31.2** | 19.6 |  | 24 | 190 |
|  | water | 0 | 100 | 0.0 | 8.8 | 36.5 |  |  |  |  | **15.1** | 19.1 |  | 24 | 174 |
|  | Mia | 50 | 0 | 100 | 100 | 98.6 |  |  |  |  | **99.5** | 0.8 | **0.11** | 24 | 199 |
|  | Mia | 50 | 15 | 96.4 | 89.9 | 96.7 |  |  |  |  | **94.3** | 3.9 | **0.07** | 24 | 197 |
|  | Mia | 50 | 25 | 93.0 | 95.9 | 96.4 |  |  |  |  | **95.1** | 1.9 | **1.78E-02** | 24 | 185 |
|  | Mia | 50 | 50 | 94.2 | 78.2 | 100 |  |  |  |  | **90.8** | 11.3 | **1.37E-02** | 24 | 172 |
|  | Mia | 50 | 75 | 63.0 | 43.2 | 87.6 |  |  |  |  | **64.6** | 22.3 | **0.12** | 24 | 157 |
|  | Mia | 50 | 100 | 13.1 | 29.4 | 73.4 |  |  |  |  | **38.6** | 31.1 | **0.34** | 24 | 146 |
| ***sod-5***  ***(tm1146)*** | water | 0 | 0 | 97.3 | 91.3 | 83.6 |  |  |  |  | **90.7** | 6.9 |  | 24 | 197 |
|  | water | 0 | 15 | 83.0 | 68.3 | 54.5 |  |  |  |  | **68.6** | 14.3 |  | 24 | 192 |
|  | water | 0 | 25 | 79.3 | 50.6 | 56.9 |  |  |  |  | **62.3** | 15.1 |  | 24 | 205 |
|  | water | 0 | 50 | 58.8 | 40.6 | 56.5 |  |  |  |  | **52.0** | 9.9 |  | 24 | 218 |
|  | water | 0 | 75 | 26.0 | 7.4 | 37.1 |  |  |  |  | **23.5** | 15.0 |  | 24 | 192 |
|  | water | 0 | 100 | 0.0 | 4.2 | 40.1 |  |  |  |  | **14.8** | 22.1 |  | 24 | 176 |
|  | Mia | 50 | 0 | 100 | 99.0 | 98.2 |  |  |  |  | **99.1** | 0.9 | **0.17** | 24 | 177 |
|  | Mia | 50 | 15 | 98.4 | 96.2 | 98.9 |  |  |  |  | **97.8** | 1.5 | **0.07** | 24 | 188 |
|  | Mia | 50 | 25 | 98.6 | 97.7 | 94.5 |  |  |  |  | **96.9** | 2.2 | **0.06** | 24 | 233 |
|  | Mia | 50 | 50 | 92.2 | 77.4 | 91.2 |  |  |  |  | **86.9** | 8.3 | **1.01E-02** | 24 | 186 |
|  | Mia | 50 | 75 | 58.8 | 30.5 | 87.5 |  |  |  |  | **58.9** | 28.5 | **0.15** | 24 | 164 |
|  | Mia | 50 | 100 | 4.5 | 16.6 | 75.6 |  |  |  |  | **32.2** | 38.0 | **0.54** | 24 | 166 |
| ***ctl-(ok1242)*** | water | 0 | 0 | 95.9 | 97.6 | 98.6 |  |  |  |  | **97.4** | 1.4 |  | 24 | 219 |
|  | water | 0 | 15 | 77.1 | 83.2 | 82.5 |  |  |  |  | **80.9** | 3.3 |  | 24 | 211 |
|  | water | 0 | 25 | 71.3 | 71.7 | 83.0 |  |  |  |  | **75.3** | 6.6 |  | 24 | 182 |
|  | water | 0 | 50 | 52.8 | 39.8 | 70.1 |  |  |  |  | **54.2** | 15.2 |  | 24 | 186 |
|  | water | 0 | 75 | 43.4 | 13.1 | 51.9 |  |  |  |  | **36.2** | 20.4 |  | 24 | 159 |
|  | water | 0 | 100 | 37.1 | 2.1 | 50.7 |  |  |  |  | **29.9** | 25.1 |  | 24 | 186 |
|  | Mia | 50 | 0 | 91.5 | 97.9 | 96.4 |  |  |  |  | **95.2** | 3.3 | **0.39** | 24 | 196 |
|  | Mia | 50 | 15 | 86.6 | 84.0 | 90.1 |  |  |  |  | **86.9** | 3.1 | **0.08** | 24 | 191 |
|  | Mia | 50 | 25 | 82.1 | 56.5 | 77.1 |  |  |  |  | **71.9** | 13.6 | **0.72** | 24 | 235 |
|  | Mia | 50 | 50 | 65.8 | 30.6 | 65.5 |  |  |  |  | **53.9** | 20.2 | **0.99** | 24 | 181 |
|  | Mia | 50 | 75 | 59.6 | 14.6 | 56.8 |  |  |  |  | **43.7** | 25.2 | **0.71** | 24 | 197 |
|  | Mia | 50 | 100 | 40.2 | 2.1 | 31.0 |  |  |  |  | **24.5** | 19.9 | **0.78** | 24 | 165 |
| ***ctl-2***  ***(ok1137)*** | water | 0 | 0 | 99.0 | 86.9 | 98.9 |  |  |  |  | **94.9** | 6.9 |  | 24 | 164 |
|  | water | 0 | 15 | 92.9 | 83.4 | 89.9 |  |  |  |  | **88.7** | 4.9 |  | 24 | 177 |
|  | water | 0 | 25 | 83.4 | 69.9 | 66.2 |  |  |  |  | **73.2** | 9.1 |  | 24 | 144 |
|  | water | 0 | 50 | 72.3 | 64.4 | 63.8 |  |  |  |  | **66.8** | 4.7 |  | 24 | 145 |
|  | water | 0 | 75 | 57.9 | 39.8 | 60.0 |  |  |  |  | **52.6** | 11.1 |  | 24 | 168 |
|  | water | 0 | 100 | 55.7 | 9.4 | 33.8 |  |  |  |  | **32.9** | 23.2 |  | 24 | 150 |
|  | Mia | 50 | 0 | 94.0 | 97.8 | 97.9 |  |  |  |  | **96.6** | 2.2 | **0.72** | 24 | 175 |
|  | Mia | 50 | 15 | 100 | 94.4 | 100 |  |  |  |  | **98.1** | 3.2 | **0.06** | 24 | 184 |
|  | Mia | 50 | 25 | 100 | 88.3 | 91.7 |  |  |  |  | **93.3** | 6.0 | **3.97E-02** | 24 | 173 |
|  | Mia | 50 | 50 | 99.0 | 90.5 | 91.4 |  |  |  |  | **93.6** | 4.7 | **2.23E-03** | 24 | 177 |
|  | Mia | 50 | 75 | 95.1 | 80.8 | 91.0 |  |  |  |  | **89.0** | 7.3 | **1.28E-02** | 24 | 167 |
|  | Mia | 50 | 100 | 95.8 | 47.9 | 84.3 |  |  |  |  | **76.0** | 25.0 | **0.09** | 24 | 132 |
| ***ctl-3***  ***(ok2042)*** | water | 0 | 0 | 96.1 | 98.9 | 100 |  |  |  |  | **98.3** | 2.0 |  | 24 | 227 |
|  | water | 0 | 15 | 84.9 | 97.4 | 98.8 |  |  |  |  | **93.7** | 7.6 |  | 24 | 209 |
|  | water | 0 | 25 | 92.3 | 93.3 | 87.2 |  |  |  |  | **90.9** | 3.3 |  | 24 | 226 |
|  | water | 0 | 50 | 63.0 | 93.8 | 82.0 |  |  |  |  | **79.6** | 15.5 |  | 24 | 224 |
|  | water | 0 | 75 | 57.5 | 77.6 | 83.5 |  |  |  |  | **72.9** | 13.6 |  | 24 | 208 |
|  | water | 0 | 100 | 36.1 | 66.9 | 53.9 |  |  |  |  | **52.3** | 15.5 |  | 24 | 175 |
|  | Mia | 50 | 0 | 98.8 | 100 | 99.2 |  |  |  |  | **99.3** | 0.6 | **0.49** | 24 | 226 |
|  | Mia | 50 | 15 | 100 | 98.6 | 100 |  |  |  |  | **99.5** | 0.8 | **0.32** | 24 | 210 |
|  | Mia | 50 | 25 | 100 | 97.9 | 100 |  |  |  |  | **99.3** | 1.2 | **3.48E-02** | 24 | 207 |
|  | Mia | 50 | 50 | 90.2 | 94.9 | 94.8 |  |  |  |  | **93.3** | 2.7 | **0.27** | 24 | 240 |
|  | Mia | 50 | 75 | 84.5 | 91.2 | 92.2 |  |  |  |  | **89.3** | 4.2 | **0.16** | 24 | 189 |
|  | Mia | 50 | 100 | 62.1 | 83.3 | 81.5 |  |  |  |  | **75.6** | 11.8 | **0.11** | 24 | 173 |
| ***prdx-2***  ***(gk169)*** | water | 0 | 0 | 100 | 100 | 94.8 |  |  |  |  | **98.3** | 3.0 |  | 24 | 239 |
|  | water | 0 | 15 | 89.8 | 88.6 | 79.7 |  |  |  |  | **86.0** | 5.5 |  | 24 | 222 |
|  | water | 0 | 25 | 66.8 | 58.0 | 73.8 |  |  |  |  | **66.2** | 7.9 |  | 24 | 235 |
|  | water | 0 | 50 | 35.1 | 43.1 | 48.4 |  |  |  |  | **42.2** | 6.7 |  | 24 | 232 |
|  | water | 0 | 75 | 13.9 | 11.3 | 18.0 |  |  |  |  | **14.4** | 3.4 |  | 24 | 205 |
|  | water | 0 | 100 | 3.7 | 9.0 | 0.0 |  |  |  |  | **4.2** | 4.5 |  | 24 | 206 |
|  | Mia | 50 | 0 | 98.8 | 95.6 | 100 |  |  |  |  | **98.1** | 2.3 | **0.95** | 24 | 190 |
|  | Mia | 50 | 15 | 30.9 | 41.4 | 56.6 |  |  |  |  | **43.0** | 12.9 | **1.7E-02** | 24 | 252 |
|  | Mia | 50 | 25 | 20.9 | 28.3 | 2.5 |  |  |  |  | **17.2** | 13.3 | **9.56E-03** | 24 | 186 |
|  | Mia | 50 | 50 | 9.1 | 10.5 | 0.0 |  |  |  |  | **6.5** | 5.7 | **2.36E-03** | 24 | 225 |
|  | Mia | 50 | 75 | 1.9 | 1.3 | 0.0 |  |  |  |  | **1.0** | 0.9 | **1.51E-02** | 24 | 216 |
|  | Mia | 50 | 100 | 0.0 | 1.1 | 0.0 |  |  |  |  | **0.4** | 0.7 | **0.28** | 24 | 190 |
| ***prdx-3***  ***(gk529)*** | water | 0 | 0 | 91.8 | 95.1 | 95.6 |  |  |  |  | **94.2** | 2.1 |  | 24 | 226 |
|  | water | 0 | 15 | 88.3 | 93.2 | 69.7 |  |  |  |  | **83.7** | 12.4 |  | 24 | 224 |
|  | water | 0 | 25 | 89.4 | 84.6 | 65.7 |  |  |  |  | **79.9** | 12.5 |  | 24 | 215 |
|  | water | 0 | 50 | 63.9 | 76.2 | 62.4 |  |  |  |  | **67.5** | 7.6 |  | 24 | 206 |
|  | water | 0 | 75 | 46.9 | 54.2 | 56.7 |  |  |  |  | **52.6** | 5.1 |  | 24 | 204 |
|  | water | 0 | 100 | 34.0 | 24.4 | 41.8 |  |  |  |  | **33.4** | 8.7 |  | 24 | 179 |
|  | Mia | 50 | 0 | 98.0 | 99.0 | 100 |  |  |  |  | **99.0** | 1.0 | **3.76E-02** | 24 | 187 |
|  | Mia | 50 | 15 | 96.5 | 98.2 | 100 |  |  |  |  | **98.2** | 1.8 | **0.18** | 24 | 216 |
|  | Mia | 50 | 25 | 98.9 | 97.2 | 100 |  |  |  |  | **98.7** | 1.4 | **0.12** | 24 | 199 |
|  | Mia | 50 | 50 | 94.1 | 97.0 | 96.3 |  |  |  |  | **95.8** | 1.5 | **1.98E-02** | 24 | 224 |
|  | Mia | 50 | 75 | 87.8 | 86.5 | 98.9 |  |  |  |  | **91.1** | 6.8 | **1.93E-03** | 24 | 215 |
|  | Mia | 50 | 100 | 80.9 | 84.6 | 72.7 |  |  |  |  | **79.4** | 6.1 | **2.62E-03** | 24 | 183 |

Summary of all stress resistance assays performed in Figure 1D and 2B. The treatments, water or Mianserin (50 µM), with their indicated concentrations (conc.) were added on day 1 of adulthood. Paraquat (PQ) was added in the concentration range of 0 to 100 mM on day 5 and survival after PQ [%] was calculated 24 h later. Mean and standard deviation (S.D.) of survival after PQ [%] were calculated from 3 to 7 independent experiments (expts.). P-values were calculated between water and Mianserin treatments at the same PQ conc., using *t-test*. The total number of wells and animals from which data were collected are indicated.

**Table S4: Survival data for paraquat stress resistance assays. Related to Figure 3F.**

| **Strain** | **Treatment** | **Conc. [µM]** | **PQ conc. (mM)** | **Survival after PQ [%]**  **(expt. 1)** | **Survival after PQ [%]**  **(expt. 2)** | **Survival after PQ [%]**  **(expt. 3)** | **Survival after PQ [%]**  **(expt. 4)** | **Mean,**  **Survival after PQ [%]** | **S.D.,**  **Survival after PQ [%]** | **P-value** | **No. of wells** | **Total no. of animals** |
| --- | --- | --- | --- | --- | --- | --- | --- | --- | --- | --- | --- | --- |
| **N2** | water | 0 | 0 | 98.2 | 100 | 100 | 100 | 99.6 | 0.9 |  | 32 | 230 |
|  | water | 0 | 25 | 95.3 | 93.2 | 94.3 | 93.8 | 94.1 | 0.9 |  | 32 | 276 |
|  | water | 0 | 50 | 81.6 | 87.2 | 78.9 | 84.2 | 83.0 | 3.5 |  | 32 | 289 |
|  | water | 0 | 75 | 81.6 | 65.3 | 78.9 | 77.9 | 75.9 | 7.3 |  | 32 | 233 |
|  | water | 0 | 100 | 65.2 | 55.5 | 67.5 | 63.1 | 62.8 | 5.2 |  | 32 | 220 |
|  | water | 0 | 150 |  | 9.9 | 64.5 | 38.9 | 37.8 | 27.3 |  | 24 | 295 |
|  | Mia | 50 | 0 | 95.1 | 100 | 100 | 100 | 98.8 | 2.5 | **0.58** | 32 | 256 |
|  | Mia | 50 | 25 | 97.5 | 97.9 | 96.7 | 95.6 | 96.9 | 1.0 | **6.10E-03** | 32 | 223 |
|  | Mia | 50 | 50 | 96.7 | 100 | 95.9 | 91.9 | 96.1 | 3.3 | **1.64E-03** | 32 | 261 |
|  | Mia | 50 | 75 | 93.3 | 87.1 | 95.5 | 93.6 | 92.4 | 3.7 | **1.27E-02** | 32 | 242 |
|  | Mia | 50 | 100 | 93.4 | 79.5 | 98.0 | 97.1 | 92.0 | 8.6 | **2.21E-03** | 32 | 193 |
|  | Mia | 50 | 150 |  | 28.0 | 90.5 | 68.6 | 62.4 | 31.7 | **0.37** | 24 | 183 |
| ***unc-2***  ***(e55)*** | water | 0 | 0 | 95.1 | 100 | 100 | 100 | 98.8 | 2.4 |  | 32 | 248 |
|  | water | 0 | 25 | 89.3 | 96.4 | 100 | 96.1 | 95.5 | 4.5 |  | 32 | 221 |
|  | water | 0 | 50 | 96.9 | 96.4 | 100 | 97.0 | 97.6 | 1.6 |  | 32 | 251 |
|  | water | 0 | 75 | 94.1 | 90.9 | 80.0 | 93.0 | 89.5 | 6.5 |  | 32 | 200 |
|  | water | 0 | 100 | 90.6 | 85.7 | 97.1 | 85.2 | 89.7 | 5.5 |  | 32 | 204 |
|  | water | 0 | 150 |  | 72.6 | 95.8 | 68.0 | 78.8 | 14.9 |  | 24 | 222 |
|  | Mia | 50 | 0 | 100 | 100 | 100 | 97.5 | 99.4 | 1.3 | **0.68** | 32 | 224 |
|  | Mia | 50 | 25 | 94.7 | 100 | 93.8 | 98.2 | 96.7 | 2.9 | **0.67** | 32 | 237 |
|  | Mia | 50 | 50 | 95.2 | 96.6 | 94.4 | 96.8 | 95.8 | 1.1 | **0.12** | 32 | 239 |
|  | Mia | 50 | 75 | 89.8 | 89.0 | 96.4 | 78.5 | 88.4 | 7.4 | **0.83** | 32 | 240 |
|  | Mia | 50 | 100 | 91.6 | 77.1 | 71.7 | 83.1 | 80.9 | 8.5 | **0.14** | 32 | 160 |
|  | Mia | 50 | 150 |  | 27.2 | 73.3 | 21.1 | 40.5 | 28.5 | **0.12** | 24 | 137 |
| ***unc-18***  ***(e18)*** | water | 0 | 0 | 95.7 | 100 | 100 | 100 | 98.9 | 2.1 |  | 31 | 141 |
|  | water | 0 | 25 | 100 | 97.5 | 71.4 | 98.4 | 91.8 | 13.6 |  | 31 | 176 |
|  | water | 0 | 50 | 100 | 89.8 | 80.0 | 96.7 | 91.6 | 8.8 |  | 31 | 151 |
|  | water | 0 | 75 | 70.2 | 74.0 | 69.4 | 92.3 | 76.5 | 10.7 |  | 31 | 156 |
|  | water | 0 | 100 | 73.5 | 43.0 | 92.4 | 86.7 | 73.9 | 22.1 |  | 31 | 141 |
|  | water | 0 | 150 |  | 41.4 | 71.4 | 58.5 | 57.1 | 15.1 |  | 23 | 141 |
|  | Mia | 50 | 0 | 100 | 100 | 100 | 100 | 100 | 0.0 | **0.39** | 32 | 154 |
|  | Mia | 50 | 25 | 100 | 100 | 100 | 100 | 100 | 0.0 | **0.32** | 32 | 155 |
|  | Mia | 50 | 50 | 92.5 | 89.9 | 80.0 | 96.8 | 89.8 | 7.1 | **0.76** | 32 | 154 |
|  | Mia | 50 | 75 | 66.5 | 63.4 | 77.4 | 58.8 | 66.5 | 7.9 | **0.19** | 32 | 148 |
|  | Mia | 50 | 100 | 68.8 | 45.6 | 65.3 | 66.9 | 61.6 | 10.8 | **0.37** | 32 | 129 |
|  | Mia | 50 | 150 |  | 21.9 | 63.3 | 35.7 | 40.3 | 21.1 | **0.33** | 24 | 105 |
| ***unc-26***  ***(e205)*** | water | 0 | 0 | 100 | 100 | 98.4 | 100 | 99.6 | 0.8 |  | 32 | 252 |
|  | water | 0 | 25 | 97.2 | 100 | 100 | 97.9 | 98.8 | 1.4 |  | 32 | 269 |
|  | water | 0 | 50 | 97.9 | 100 | 100 | 100 | 99.5 | 1.0 |  | 32 | 239 |
|  | water | 0 | 75 | 88.2 | 91.7 | 84.8 | 93.4 | 89.5 | 3.8 |  | 32 | 253 |
|  | water | 0 | 100 | 74.9 | 93.3 | 78.2 | 73.7 | 80.0 | 9.1 |  | 32 | 226 |
|  | water | 0 | 150 |  | 61.5 | 80.9 | 79.8 | 74.1 | 10.9 |  | 24 | 260 |
|  | Mia | 50 | 0 | 100 | 100 | 100 | 100 | 100 | 0.0 | **0.39** | 32 | 227 |
|  | Mia | 50 | 25 | 97.9 | 100 | 97.5 | 98.6 | 98.5 | 1.1 | **0.77** | 32 | 282 |
|  | Mia | 50 | 50 | 88.5 | 94.4 | 97.9 | 94.6 | 93.8 | 3.9 | **0.06** | 32 | 258 |
|  | Mia | 50 | 75 | 94.0 | 70.0 | 90.5 | 76.6 | 82.8 | 11.4 | **0.33** | 32 | 247 |
|  | Mia | 50 | 100 | 67.6 | 63.0 | 81.4 | 42.9 | 63.7 | 16.0 | **0.14** | 32 | 186 |
|  | Mia | 50 | 150 |  | 12.5 | 86.7 | 18.7 | 39.3 | 41.1 | **0.28** | 24 | 164 |
| ***snt-1***  ***(md290)*** | water | 0 | 0 | 94.2 | 100 | 100 | 98.9 | 98.3 | 2.8 |  | 32 | 273 |
|  | water | 0 | 25 | 84.9 | 92.9 | 93.4 | 99.0 | 92.5 | 5.8 |  | 32 | 264 |
|  | water | 0 | 50 | 82.5 | 88.8 | 89.7 | 94.7 | 88.9 | 5.0 |  | 32 | 304 |
|  | water | 0 | 75 | 82.3 | 83.0 | 85.9 | 92.4 | 85.9 | 4.6 |  | 32 | 292 |
|  | water | 0 | 100 | 72.6 | 56.6 | 74.2 | 70.7 | 68.5 | 8.1 |  | 32 | 260 |
|  | water | 0 | 150 |  | 47.5 | 70.1 | 33.0 | 50.2 | 18.7 |  | 24 | 261 |
|  | Mia | 50 | 0 | 100 | 100 | 100 | 100 | 100 | 0.0 | **0.30** | 32 | 270 |
|  | Mia | 50 | 25 | 95.3 | 84.9 | 96.0 | 97.5 | 93.4 | 5.8 | **0.84** | 32 | 271 |
|  | Mia | 50 | 50 | 79.5 | 90.8 | 98.8 | 88.0 | 89.3 | 8.0 | **0.94** | 32 | 274 |
|  | Mia | 50 | 75 | 76.9 | 68.4 | 86.2 | 60.5 | 73.0 | 11.0 | **0.10** | 32 | 249 |
|  | Mia | 50 | 100 | 69.8 | 61.4 | 81.3 | 70.0 | 70.6 | 8.2 | **0.73** | 32 | 182 |
|  | Mia | 50 | 150 |  | 54.6 | 64.4 | 19.5 | 46.2 | 23.6 | **0.83** | 24 | 188 |
| ***snb-1***  ***(md247)*** | water | 0 | 0 | 90.9 | 88.4 | 99.0 |  | 92.8 | 5.5 |  | 24 | 232 |
|  | water | 0 | 25 | 49.2 | 66.5 | 64.8 |  | 60.2 | 9.5 |  | 24 | 231 |
|  | water | 0 | 50 | 21.2 | 29.8 | 48.6 |  | 33.2 | 14.0 |  | 24 | 223 |
|  | water | 0 | 75 | 12.9 | 17.6 | 62.5 |  | 31.0 | 27.4 |  | 24 | 249 |
|  | water | 0 | 100 | 9.6 | 20.0 | 26.6 |  | 18.7 | 8.5 |  | 24 | 208 |
|  | water | 0 | 150 | 2.2 | 12.4 | 8.6 |  | 10.5 | 2.7 |  | 24 | 225 |
|  | Mia | 50 | 0 | 93.5 | 98.0 | 93.5 |  | 95.0 | 2.6 | **0.58** | 24 | 210 |
|  | Mia | 50 | 25 | 8.8 | 25.3 | 49.7 |  | 27.9 | 20.6 | **0.10** | 24 | 233 |
|  | Mia | 50 | 50 | 0.0 | 19.2 | 48.6 |  | 22.6 | 24.5 | **0.56** | 24 | 226 |
|  | Mia | 50 | 75 | 1.8 | 8.4 | 28.5 |  | 12.9 | 13.9 | **0.38** | 24 | 221 |
|  | Mia | 50 | 100 | 0.0 | 14.3 | 33.6 |  | 16.0 | 16.9 | **0.82** | 24 | 226 |
|  | Mia | 50 | 150 | 0.0 | 4.1 | 1.7 |  | 2.9 | 1.7 | **0.18** | 24 | 213 |
| ***unc-10***  ***(e102)*** | water | 0 | 0 | 98.2 | 94.8 | 96.9 | 100.0 | 97.5 | 2.2 |  | 32 | 196 |
|  | water | 0 | 25 | 97.9 | 100.0 | 96.9 | 91.7 | 96.6 | 3.5 |  | 32 | 191 |
|  | water | 0 | 50 | 73.9 | 84.6 | 88.0 | 86.7 | 83.3 | 6.4 |  | 32 | 223 |
|  | water | 0 | 75 | 67.3 | 69.6 | 63.9 | 66.0 | 66.7 | 2.4 |  | 32 | 223 |
|  | water | 0 | 100 | 33.9 | 34.2 | 49.6 | 26.9 | 36.2 | 9.6 |  | 32 | 197 |
|  | water | 0 | 150 |  | 9.6 | 10.2 | 0.0 | 6.6 | 5.7 |  | 24 | 185 |
|  | Mia | 50 | 0 | 100.0 | 100.0 | 100.0 | 96.9 | 99.2 | 1.5 | **0.25** | 32 | 209 |
|  | Mia | 50 | 25 | 89.4 | 90.9 | 100.0 | 78.7 | 89.7 | 8.7 | **0.22** | 32 | 224 |
|  | Mia | 50 | 50 | 64.6 | 51.9 | 90.9 | 45.8 | 63.3 | 20.0 | **0.14** | 32 | 188 |
|  | Mia | 50 | 75 | 47.4 | 53.7 | 66.8 | 23.4 | 47.8 | 18.2 | **0.13** | 32 | 218 |
|  | Mia | 50 | 100 | 10.1 | 5.9 | 38.4 | 22.3 | 19.2 | 13.4 | **0.11** | 32 | 197 |
|  | Mia | 50 | 150 |  | 7.3 | 14.4 | 0.0 | 7.2 | 5.9 | **0.91** | 24 | 172 |
| ***unc-11***  ***(e47)*** | water | 0 | 0 | 93.8 | 100.0 | 98.2 | 100.0 | 98.0 | 2.9 |  | 32 | 260 |
|  | water | 0 | 25 | 95.4 | 94.3 | 97.8 | 98.2 | 96.4 | 1.9 |  | 32 | 273 |
|  | water | 0 | 50 | 89.9 | 90.6 | 92.4 | 95.0 | 92.0 | 2.3 |  | 32 | 240 |
|  | water | 0 | 75 | 77.0 | 91.5 | 88.5 | 87.1 | 86.0 | 6.3 |  | 32 | 268 |
|  | water | 0 | 100 | 66.8 | 80.1 | 65.1 | 70.3 | 70.6 | 6.7 |  | 32 | 247 |
|  | water | 0 | 150 |  | 35.1 | 41.6 | 28.3 | 35.0 | 6.6 |  | 24 | 271 |
|  | Mia | 50 | 0 | 93.5 | 100.0 | 100.0 | 100.0 | 98.4 | 0.8 | **0.87** | 32 | 282 |
|  | Mia | 50 | 25 | 92.9 | 95.0 | 97.1 | 98.2 | 95.8 | 1.4 | **0.70** | 32 | 285 |
|  | Mia | 50 | 50 | 86.5 | 91.2 | 94.8 | 91.9 | 91.1 | 1.8 | **0.69** | 32 | 292 |
|  | Mia | 50 | 75 | 66.1 | 83.4 | 80.4 | 54.0 | 71.0 | 13.2 | **0.11** | 32 | 262 |
|  | Mia | 50 | 100 | 26.6 | 61.4 | 59.1 | 64.0 | 52.8 | 4.8 | **0.13** | 32 | 253 |
|  | Mia | 50 | 150 |  | 8.8 | 19.6 | 14.0 | 14.1 | 4.4 | **1.46E-02** | 24 | 266 |
| ***che-12***  ***(e1812)*** | water | 0 | 0 | 96 | 93.8 | 97.9 |  | 95.9 | 2.1 |  | 24 | 190 |
|  | water | 0 | 15 | 91.6 | 70.8 | 81.1 |  | 81.2 | 10.4 |  | 24 | 224 |
|  | water | 0 | 25 | 70.1 | 53.3 | 47.6 |  | 57.0 | 11.7 |  | 24 | 207 |
|  | water | 0 | 50 | 45.9 | 12 | 34.6 |  | 30.8 | 17.3 |  | 24 | 238 |
|  | water | 0 | 75 | 28.1 | 8.2 | 9.3 |  | 15.2 | 11.2 |  | 24 | 203 |
|  | water | 0 | 100 | 7.2 | 6.6 | 9 |  | 7.6 | 1.2 |  | 24 | 213 |
|  | Mia | 50 | 0 | 93.6 | 98.9 | 98.9 |  | 97.1 | 1.0 | **0.60** | 24 | 191 |
|  | Mia | 50 | 15 | 71.2 | 91 | 77.6 |  | 79.9 | 7.2 | **0.89** | 24 | 200 |
|  | Mia | 50 | 25 | 33 | 16.6 | 17.1 |  | 22.2 | 3.1 | **1.74E-02** | 24 | 206 |
|  | Mia | 50 | 50 | 12.7 | 4.4 | 0 |  | 5.7 | 3.0 | **0.11** | 24 | 209 |
|  | Mia | 50 | 75 | 4.5 | 1.7 | 2.5 |  | 2.9 | 0.6 | **0.20** | 24 | 198 |
|  | Mia | 50 | 100 | 2.3 | 0 | 0 |  | 0.8 | 0.4 | **2.94E-03** | 24 | 219 |

Summary of all stress resistance assays performed in Figure 3F. The treatments, water or Mianserin (50 µM) at their indicated concentrations (conc.) were added on day 1 of adulthood. Paraquat (PQ) was added in the concentration range of 0 to 100 mM on day 5 and survival after PQ [%] was calculated 24 h later. Mean and standard deviation (S.D.) of survival after PQ [%] were calculated from 3 to 4 independent experiments (expts.). P-values were calculated between water and Mianserin treatments using *t-test*. The total number of wells and animals from which data were collected are indicated.

**Table S5: Summary of oxidative stress protection by serotonin antagonists. Related to Figure 3H.**

| **Strain name** | **Fold change in survival after PQ [(Drug/DMSO) -1]**  **Expt.1** | **Fold change in survival after PQ [(Drug/DMSO) -1]**  **Expt.2** | **Fold change in survival after PQ [(Drug/DMSO) -1]**  **Expt.3** | **Fold change in survival after PQ [(Drug/DMSO) -1]**  **Expt.4** | **Fold change in survival after PQ [(Drug/DMSO) -1]**  **Expt.5** | **Fold change in survival after PQ [(Drug/DMSO) -1]**  **Expt.6** | **Fold change in survival after PQ [(Drug/DMSO) -1]**  **Expt.7** | **Mean,**  **Fold change in survival after PQ** | **S.D.,**  **Fold change in survival after PQ** | **P-value** |
| --- | --- | --- | --- | --- | --- | --- | --- | --- | --- | --- |
| **Dihydroergotamine 88 µM** | | | | | | | | | | |
| **N2** | 0.62 | 0.70 | 0.79 | 0.19 | 1.75 | 1.43 |  | **0.91** | 0.57 |  |
| ***prdx-2(gk169)*** | 0.22 |  | 0.01 |  | -0.57 |  |  | **-0.11** | 0.41 | **2.26E-02** |
| ***sod-1(tm776)*** | 0.09 | 0.49 | 0.24 |  | -0.20 |  |  | **0.16** | 0.29 | **2.57E-02** |
| ***unc-26(e205)*** | -0.07 |  |  |  | 0.17 | -0.05 |  | **0.02** | 0.13 | **1.09E-02** |
| ***snt-1(md290)*** | -0.01 |  |  | 0.04 |  | -0.14 |  | **-0.03** | 0.09 | **8.95E-03** |
| ***ctl-1(ok1242)*** | 0.41 |  | 1.10 | 0.27 |  |  |  | **0.60** | 0.45 | **0.40** |
| ***gst-4(tm3294)*** | 0.46 |  | 0.49 | 0.35 |  |  |  | **0.43** | 0.07 | **0.09** |
| **Metergoline 33 µM** | | | | | | | | | | |
| **N2** | 0.54 | 0.57 | 0.68 | 0.94 | 1.24 | 1.67 |  | **0.94** | 0.44 |  |
| ***prdx-2(gk169)*** |  | -0.44 |  | -0.05 |  | -0.15 |  | **-0.21** | 0.20 | **1.09E-03** |
| ***sod-1(tm776)*** |  | -0.27 | 0.21 | 0.25 |  | 0.15 |  | **0.09** | 0.24 | **4.54E-03** |
| ***unc-26(e205)*** | -0.02 | 0.06 |  |  |  | 0.35 |  | **0.13** | 0.19 | **6.57E-03** |
| ***snt-1(md290)*** | -0.14 | -0.14 |  |  | -0.07 |  |  | **-0.12** | 0.04 | **1.95E-03** |
| ***ctl-1(ok1242)*** |  | 0.26 |  | 1.22 | 0.01 |  |  | **0.50** | 0.64 | **0.36** |
| ***gst-4(tm3294)*** |  | 0.21 |  | 0.86 | 0.17 |  |  | **0.41** | 0.39 | **0.13** |
| **Amperozide 13 µM** | | | | | | | | | | |
| **N2** | 0.93 | 0.74 | 0.99 | 0.92 | 2.49 | 0.89 |  | **1.16** | 0.66 |  |
| ***prdx-2(gk169)*** | -0.19 |  | 0.16 |  | -0.62 |  |  | **-0.22** | 0.39 | **6.66E-03** |
| ***sod-1(tm776)*** | -0.25 | 0.50 | 0.40 |  | 0.36 |  |  | **0.25** | 0.34 | **2.19E-02** |
| ***unc-26(e205)*** | 0.00 |  |  |  | -0.18 | -0.07 |  | **-0.08** | 0.09 | **5.14E-03** |
| ***snt-1(md290)*** | 0.00 |  |  | 0.00 |  | -0.26 |  | **-0.09** | 0.15 | **4.59E-03** |
| ***ctl-1(ok1242)*** | 0.40 |  | 1.27 | 0.01 |  |  |  | **0.56** | 0.65 | **0.26** |
| ***gst-4(tm3294)*** | 0.26 |  | 0.64 | 0.02 |  |  |  | **0.31** | 0.31 | **3.40E-02** |
| **Methiothepin 10 µM** | | | | | | | | | | |
| **N2** | 0.80 | 1.08 | 0.95 | 0.36 | 0.77 | 2.94 | 1.39 | **1.19** | 0.89 |  |
| ***prdx-2(gk169)*** | -0.47 |  | -0.24 |  |  | -0.69 |  | **-0.47** | 0.31 | **1.49E-03** |
| ***sod-1(tm776)*** | -0.54 | 0.22 | 0.16 |  |  | -0.02 |  | **-0.04** | 0.12 | **8.17E-03** |
| ***unc-26(e205)*** | -0.16 |  |  |  |  | -0.11 | -0.03 | **-0.10** | 0.06 | **6.30E-03** |
| ***snt-1(md290)*** | -0.33 |  |  | -0.23 | -0.21 |  | -0.07 | **-0.21** | 0.08 | **4.18E-03** |
| ***ctl-1(ok1242)*** | 0.35 |  | 0.27 | 0.34 | 0.21 |  |  | **0.29** | 0.07 | **2.95E-02** |
| ***gst-4(tm3294)*** | 0.24 |  | 1.35 | -0.10 | 0.40 |  |  | **0.47** | 0.74 | **0.15** |
| **Ketanserin 176 µM** | | | | | | | | | | |
| **N2** | 0.63 | 0.59 | 1.13 | 1.38 | 0.42 | 1.71 |  | **0.98** | 0.51 |  |
| ***prdx-2(gk169)*** |  | -0.07 |  | -0.04 |  | -0.22 |  | **-0.11** | 0.10 | **2.83E-03** |
| ***sod-1(tm776)*** |  | -0.36 | -0.14 | 0.21 |  | -0.14 |  | **-0.11** | 0.23 | **2.34E-03** |
| ***unc-26(e205)*** | -0.30 | 0.00 |  |  |  | 0.15 |  | **-0.05** | 0.23 | **4.23E-03** |
| ***snt-1(md290)*** | -0.39 | -0.11 |  |  | -0.22 |  |  | **-0.24** | 0.14 | **1.43E-03** |
| ***ctl-1(ok1242)*** |  | 0.50 |  | 0.33 | 0.19 |  |  | **0.34** | 0.16 | **2.95E-02** |
| ***gst-4(tm3294)*** |  | 0.39 |  | 0.77 | 0.35 |  |  | **0.51** | 0.23 | **0.10** |
| **Mirtazapine 50 µM** | | | | | | | | | | |
| **N2** | 0.8 | 0.7 | 1.1 | 0.4 | 1.0 | 0.8 | 1.5 | **0.89** | 0.35 |  |
| ***prdx-2(gk169)*** | 0.3 |  | -0.2 |  |  | -0.2 |  | **-0.07** | 0.29 | **8.17E-03** |
| ***sod-1(tm776)*** | -0.6 | 0.0 | -0.2 |  |  | -0.2 |  | **-0.22** | 0.25 | **2.35E-04** |
| ***unc-26(e205)*** | 0.1 |  |  |  |  | 0.1 | 0.0 | **0.07** | 0.05 | **6.59E-04** |
| ***snt-1(md290)*** | -0.1 |  |  | -0.1 | -0.1 |  | 0.0 | **-0.06** | 0.05 | **3.10E-04** |
| ***ctl-1(ok1242)*** | 0.3 |  | 0.1 | -0.4 | 0.1 |  |  | **0.02** | 0.29 | **2.72E-03** |
| ***gst-4(tm3294)*** | 0.8 |  | 1.4 | 0.2 | 0.2 |  |  | **0.67** | 0.59 | **0.53** |
| **LY-165,163 33/PAPP µM** | | | | | | | | | | |
| **N2** | 0.48 | 0.49 | 1.00 | 0.94 | 0.53 | 1.40 |  | **0.81** | 0.37 |  |
| ***prdx-2(gk169)*** |  | -0.61 |  | -0.60 |  | -0.37 |  | **-0.53** | 0.13 | **1.18E-04** |
| ***sod-1(tm776)*** |  | -1.00 | -0.28 | -0.27 |  | -0.34 |  | **-0.47** | 0.35 | **9.69E-04** |
| ***unc-26(e205)*** | -0.01 | -0.02 |  |  |  | -0.06 |  | **-0.03** | 0.03 | **2.45E-03** |
| ***snt-1(md290)*** | 0.39 | 0.09 |  |  | -0.03 |  |  | **0.15** | 0.22 | **1.37E-02** |
| ***ctl-1(ok1242)*** |  | 0.27 |  | 0.07 | -0.14 |  |  | **0.07** | 0.20 | **6.54E-03** |
| ***gst-4(tm3294)*** |  | 0.81 |  | 2.34 | 0.28 |  |  | **1.14** | 1.07 | **0.65** |
| **Mianserin 50 µM** | | | | | | | | | | |
| **N2** | 1.10 | 1.11 | 1.18 | 0.53 | 3.24 | 1.60 |  | **1.46** | 0.94 |  |
| ***prdx-2(gk169)*** | -1.00 |  | -0.51 |  | -0.90 |  |  | **-0.81** | 0.26 | **1.26E-03** |
| ***sod-1(tm776)*** | -1.00 | -1.00 | -0.37 |  | -0.78 |  |  | **-0.79** | 0.30 | **5.88E-03** |
| ***unc-26(e205)*** | -0.22 |  |  |  | -0.03 | -0.10 |  | **-0.12** | 0.09 | **7.95E-03** |
| ***snt-1(md290)*** | -0.09 |  |  | -0.16 |  | -0.23 |  | **-0.16** | 0.07 | **8.32E-03** |
| ***ctl-1(ok1242)*** | 0.21 |  | 0.83 | -0.09 |  |  |  | **0.32** | 0.47 | **1.28E-03** |
| ***gst-4(tm3294)*** | 0.76 |  | 1.86 | 0.22 |  |  |  | **0.95** | 0.83 | **0.44** |

Summary of all stress resistance assays performed in Figure 3H. The treatments, DMSO or serotonin antagonists with their indicated concentrations (conc.) were added on day 1 of adulthood. Paraquat (PQ) (N2 : 100mM PQ; *prdx-2(gk169)*: 50mM PQ; *sod-1(tm776)*: 25mM PQ; *unc-26(e205)*: 100mM PQ; *snt-1(md290)*: 100mM PQ; ctl-1(ok1242: 100mM PQ; gst-4(tm3294): 100mM PQ) was added on day 5 and survival after PQ [%] was calculated 24 h later. Mean and standard deviation (S.D.) of survival after PQ [%] were calculated from 3 to 7 independent experiments (expts.). P-values were calculated between N2 and mutant strains for fold change values with indicated small molecule treatments, using *t-test*.

**Table S6: Summary of fluorescence intensity quantification for P*gst-4*::GFP reporter after paraquat treatment. Related to Figure 4A.**

| **Strain** | **Treatment, 8h** | **Mean, Fluorescence intensity [A.U]** | **S. D., Fluorescence intensity [A.U]** | **Fold change**  **[PQ/water]** | **P-value** | **Mean Fold change**  **[PQ/water]** |
| --- | --- | --- | --- | --- | --- | --- |
| wt;  P*gst-4*::GFP | water | 0.030 | 0.012 |  |  |  |
|  | water | 0.073 | 0.030 |  |  |  |
|  | water | 0.032 | 0.014 |  |  |  |
|  | water | 0.028 | 0.010 |  |  |  |
|  | Paraquat 100mM | 0.061 | 0.031 | 2.041 | **3.38E-11** |  |
|  | Paraquat 100mM | 0.156 | 0.059 | 2.130 | **6.18E-19** |  |
|  | Paraquat 100mM | 0.082 | 0.039 | 2.556 | **3.26E-11** |  |
|  | Paraquat 100mM | 0.069 | 0.032 | 2.442 | **2.75E-10** | **2.3** |
| unc-26 (e205);  P*gst-4*::GFP | water | 0.021 | 0.008 |  |  |  |
|  | water | 0.045 | 0.013 |  |  |  |
|  | water | 0.019 | 0.007 |  |  |  |
|  | water | 0.017 | 0.005 |  |  |  |
|  | Paraquat 100mM | 0.079 | 0.038 | 3.767 | **1.73E-02** |  |
|  | Paraquat 100mM | 0.123 | 0.053 | 2.715 | **2.85E-14** |  |
|  | Paraquat 100mM | 0.064 | 0.046 | 3.337 | **2.14E-05** |  |
|  | Paraquat 100mM | 0.055 | 0.032 | 3.264 | **2.81E-08** | **3.3** |
| snt-1(md290);  P*gst-4*::GFP | water | 0.024 | 0.006 |  |  |  |
|  | water | 0.018 | 0.006 |  |  |  |
|  | water | 0.015 | 0.006 |  |  |  |
|  | water | 0.025 | 0.007 |  |  |  |
|  | Paraquat 100mM | 0.075 | 0.062 | 3.133 | **2.86E-03** |  |
|  | Paraquat 100mM | 0.053 | 0.050 | 2.944 | **3.15E-03** |  |
|  | Paraquat 100mM | 0.037 | 0.038 | 2.475 | **4.19E-03** |  |
|  | Paraquat 100mM | 0.090 | 0.040 | 3.605 | **1.37E-17** | **3.0** |

Summary of all fluorescence intensity measurements in arbitrary units (A.U) in Figure 4A. The treatments, water or 100 mM paraquat (PQ) were added to animals on day 1 of adulthood and imaged after 8 h. Mean and standard deviation (S.D.) of fluorescence intensity [A.U] were calculated from 4 independent experiments (expts.). The fold change in fluorescence intensity between PQ-treated and water-treated samples were calculated [PQ/water] from 4 independent experiments are shown. P-value was calculated between water and PQ treatments using *t-test*. The mean fold change between 4 independent experiments is shown.

**Table S7: Summary of all lifespan data for Mianserin. Related to Figure 5.**

| **Cumulative statistics** | | | | | | | **Statistics of individual expts.** | | | |
| --- | --- | --- | --- | --- | --- | --- | --- | --- | --- | --- |
| **Strain** | **Small molecule** | **No. of expts.** | **Mean lifespan [days]**  (+Mia/+water) | **change in lifespan [%]** | **S.E.M.** | **No. of animals** (+Mia/+water) | **Mean lifespan (days)**  (+Mia/+water) | **change in lifespan [%]** | **P-value** | **No. of animals** (+Mia/+water) |
| **N2** | **Mia** | 6 | **28.3/19.6** | **+46** | ±7 | 569/1000 | 27.0/21.1 | +28 | **8.99E-13** | 113/108 |
|  |  |  |  |  |  |  | 32.2/23.7 | +36 | **9.07E-18** | 104/101 |
|  |  |  |  |  |  |  | 30.9/21.1 | +46 | **3.56E-19** | 55/193 |
|  |  |  |  |  |  |  | 23.2/16.3 | +42 | **2.94E-19** | 165/172 |
|  |  |  |  |  |  |  | 27.7/15.8 | +75 | **3.87E-35** | 81/381 |
|  |  |  |  |  |  |  | 28.5/19.5 | +46 | **3.93E-08** | 51/45 |
| ***sod-1***  ***(tm776)*** | **Mia** | 6 | **27.0/24.7** | **+10** | ±2 | 607/653 | 29.2/25.5 | +15 | **2.53E-07** | 119/111 |
|  |  |  |  |  |  |  | 28.1/26.6 | +6 | **0.57** | 98/101 |
|  |  |  |  |  |  |  | 24.2/23.7 | +2 | **0.20** | 123/123 |
|  |  |  |  |  |  |  | 26.8/23.2 | +16 | **4.29E-03** | 73/131 |
|  |  |  |  |  |  |  | 27.6/25.9 | +10 | **1.04E-02** | 49/62 |
|  |  |  |  |  |  |  | 26.3/23.1 | +14 | **1.13E-05** | 145/125 |
| ***sod-1 (tm783)*** | **Mia** | 5 | **23.2/21.3** | **+10** | ±2 | 461/532 | 26.4/23.6 | +12 | **1.37E-03** | 79/98 |
|  |  |  |  |  |  |  | 22.1/21.2 | +4 | **0.06** | 89/94 |
|  |  |  |  |  |  |  | 22.8/20.0 | +14 | **2.31E-03** | 100/105 |
|  |  |  |  |  |  |  | 21.2/19.4 | +9 | **1.46E-03** | 78/103 |
|  |  |  |  |  |  |  | 23.4/22.3 | +5 | **2.75E-02** | 115/132 |
| ***ctl-1***  ***(ok1242)*** | **Mia** | 3 | **27.3/22.1** | **+23** | ±3 | 280/456 | 27.2/22.6 | +20 | **9.31E-05** | 139/258 |
|  |  |  |  |  |  |  | 27.8/23.0 | +21 | **6.23E-05** | 77/81 |
|  |  |  |  |  |  |  | 26.8/20.7 | +29 | **1.62E-05** | 64/117 |
| ***prdx-2***  ***(gk169)*** | **Mia** | 2 | **22.2/24.6** | **-10** | ±3 | 218/208 | 24.2/26.1 | -7 | **0.19** | 146/132 |
|  |  |  |  |  |  |  | 20.3/23.1 | -12 | **5.13E-03** | 72/76 |
| ***sod-2***  ***(gk257)*** | **Mia** | 4 | **27.0/20.0** | **+39** | ±12 | 428/496 | 28.1/23.9 | +17 | **6.68E-05** | 110/109 |
|  |  |  |  |  |  |  | 25.7/18.9 | +36 | **6.48E-14** | 113/135 |
|  |  |  |  |  |  |  | 28.1/15.6 | +64 | **5.24E-22** | 64/130 |
|  |  |  |  |  |  |  | 26.2/21.7 | +21 | **1.74E-09** | 141/122 |
| ***sod-3***  ***(tm760)*** | **Mia** | 4 | **27.0/17.5** | **+54** | ±3 | 488/457 | 29.5/19.9 | +48 | **6.08E-18** | 147/131 |
|  |  |  |  |  |  |  | 25.0/16.3 | +53 | **1.46E-22** | 136/124 |
|  |  |  |  |  |  |  | 26.3/16.3 | +61 | **5.16E-16** | 70/56 |
|  |  |  |  |  |  |  | 27.0/17.7 | +53 | **1.83E-26** | 135/146 |
| ***sod-4***  ***(gk101)*** | **Mia** | 4 | **27.7/19.1** | **+32** | ±7 | 382/393 | 27.5/19.6 | +40 | **1.63E-19** | 145/136 |
|  |  |  |  |  |  |  | 24.7/17.6 | +41 | **1.07E-07** | 103/87 |
|  |  |  |  |  |  |  | 31.1/20.1 | +16 | **1.87E-05** | 16/44 |
|  |  |  |  |  |  |  | 27.5/19.3 | +42 | **1.29E-18** | 118/124 |
| ***sod-5***  ***(tm1146)*** | **Mia** | 4 | **25.1/18.3** | **+40** | ±17 | 393/498 | 27.8/21.3 | +31 | **6.83E-09** | 129/100 |
|  |  |  |  |  |  |  | 20.2/18.3 | +10 | **9.86E-04** | 101/139 |
|  |  |  |  |  |  |  | 25.8/14.5 | +78 | **6.90E-17** | 54/130 |
|  |  |  |  |  |  |  | 26.8/19.2 | +40 | **2.83E-16** | 109/129 |
| ***ctl-2***  ***(ok1137)*** | **Mia** | 3 | **27.9/18.4** | **+53** | ±7 | 218/417 | 30.1/21.4 | +41 | **6.10E-19** | 102/217 |
|  |  |  |  |  |  |  | 26.4/17.2 | +53 | **1.86E-12** | 50/124 |
|  |  |  |  |  |  |  | 27.2/16.6 | +64 | **4.84E-14** | 66/76 |
| ***prdx-3***  ***(gk529)*** | **Mia** | 2 | **26.8/20.5** | **+32** | ±14 | 186/166 | 26.8/18.3 | +46 | **3.71E-24** | 142/126 |
|  |  |  |  |  |  |  | 26.8/22.7 | +18 | **1.7E-03** | 44/40 |

Summary of all lifespan experiments performed in Figure 5. N2 and mutant strains were treated with 50 µM Mianserin (Mia) on day 1 and lifespan [days] was scored until 95% of animals were dead in all tested conditions. Cumulative statistics and statistics of individual experiments are shown. Mean lifespan [days], change in lifespan [%] and S.E.M. for Mianserin-treated (+Mia) and water-treated (+water) animals from multiple, independent experiments (expts.) are shown. Change in lifespan [%] and P-values for individual experiments were calculated using the Mantel–Haenszel version of the log-rank test. Number of animals in individual experiments and all experiments combined are shown.

**Appendix S1. Extended Experimental Procedures**

**Chemicals**

Solvents used to prepare stock solutions: Paraquat were dissolved in water, Mianserin was dissolved either in water or DMSO as mentioned, Mirtazapine, Dihydroergotamine, LY-165,163/PAPP, Mirtazapine, Metergoline, Ketanserin, Methiothepin, Amperozide were dissolved in DMSO, FUDR was dissolved in S-complete, Aldicarb was dissolved in 70% ethanol. For the DPPH assays, DPPH, Mianserin, Mirtazapine and Trolox were dissolved in 100% ethanol.

**List of small molecules and chemicals used in this study with information**

| **Molecule name** | **CAS number** | **Catalog number** | **Manufacturer** |
| --- | --- | --- | --- |
| Mianserin HCl | 21535-47-7 | 0997 | Tocris |
| Mirtazapine | 85650-52-8 | M3368 | LKT Laboratories |
| Fluoxetine HCl | 59333-67-4 | F1200 | Spectrum Labs |
| Dihydroergotamine mesylate | 6190-39-2 | 0475 | Tocris/R&D systems |
| LY-165,163/PAPP | 1814-64-8 | S009 | Sigma |
| Mirtazapine | 61337-67-5 | M3368 | LKT labs |
| Metergoline | 17692-51-2 | M3668 | Sigma |
| Ketanserin tartarate | 83846-83-7 | S006 | Sigma |
| Methiothepin mesylate | 74611-28-2 | M149 | Sigma |
| Amperozide HCl | 86725-37-3 | 2746 | Tocris/R&D systems |
| Trolox | 53188-07-1 | 238813 | Sigma-Aldrich |
| Paraquat  (Methyl viologen) | 1910-42-5 | AC227320010 | Acros Organics |
| FUDR | 50-91-9 | F0503 | Sigma-Aldrich |
| DPPH | 1898-66-4 | D9132 | Sigma-Aldrich |
| DMSO | 67-68-5 | 472301 | Sigma-Aldrich |

**Strains**

Detailed descriptions of all strains used in this study are tabulated below. Mutant strains were backcrossed at least 4 times with the N2 Bristol strain. All strains were maintained as described in ([Brenner, 1974](#_ENREF_7)). CL2166 and SJ4143 males were generated by RNAi for high incidence of male (him-14) gene. CL2166 or SJ4143 males were crossed with mutant hermaphrodites to generate fluorescent reporter strains. Strain names starting with “VV” (Wormbase ID for Petrascheck, WBPerson11689) were generated in this study.

**List of mutant strains and fluorescent strains, outcrossed and used in this study.**

| **Strain name** | **Genotype** | **Number of times outcrossed** | **Gene name** | **Transgene** | **Allelle** | **Parent strain (s)** |
| --- | --- | --- | --- | --- | --- | --- |
| VV1 | *sod-1(tm776) II* | 4 | *sod-1* |  | *tm776* | FX776 |
| VV2 | *sod-1(tm783) II* | 4 | *sod-1* |  | *tm783* | FX783 |
| VV51 | *sod-3(tm760) X* | 4 | *sod-3* |  | *tm760* | FX760 |
| VV52 | *sod-4(gk101) III* | 4 | *sod-4* |  | *gk101* | VC175 |
| VV53 | *sod-5(tm1146) II* | 4 | *sod-5* |  | *tm1146* | FX1146 |
| VV54 | *sod-2(gk257) I* | 4 | *sod-2* |  | *gk257* | VC498 |
| VV71 | *dgk-1(ok1462) X* | 4 | *dgk-1* |  | *ok1462* | VC1014 |
| VV72 | *ctl-1(ok1242) II* | 4 | *ctl-1* |  | *ok1242* | RB1197 |
| VV73 | *ctl-2(ok1137) II* | 4 | *ctl-2* |  | *ok1137* | VC754 |
| VV74 | *unc-2(e55) X* | 4 | *unc-2* |  | *e55* | CB55 |
| VV75 | *unc-10(e102) X* | 4 | *unc-10* |  | *e102* | CB102 |
| VV76 | *unc-11(e47) X* | 4 | *unc-11* |  | *e47* | CB47 |
| VV77 | *unc-18(e81) X* | 4 | *unc-18* |  | *e81* | CB81 |
| VV78 | *unc-26(e205) IV* | 4 | *unc-26* |  | *e205* | CB205 |
| VV80 | *snt-1(md290) II* | 4 | *snt-1* |  | *md290* | NM204 |
| VV84 | *prdx-2 (gk169) II* | 4 | *prdx-2* |  | *gk169* | VC289 |
| VV85 | *prdx-3 (gk529) III* | 4 | *prdx-3* |  | *gk529* | VV1151 |
| VV86 | *ctl-3(ok2042) II* | 4 | *ctl-3* |  | *ok2042* | RB1653 |
| NM467 | *snb-1(md247) V* | 8 | *snb-1* |  | *md247* | NM467 |
| CB3332 | *che-12(e1812)V* | 1 | *che-12* |  | *e1812* | CB3332 |
| FX3294 | *gst-4(tm3294) IV* | 0 | *gst-4* |  | *tm3294* | FX3294 |
| SPC168 | *dvIs19 III; skn-1(lax188) IV* | 7 | *skn-1* | *dvIs19* [pAF15(gst-4::GFP::NLS] | *lax188* | SPC168 |
| CF1038 | *daf-16(mu86) I* | 11 | *daf-16* |  | *mu86* | CF1038 |
| PR802 | *osm-3(p802) IV* | 2 | *osm-3* |  | *p802* | PR802 |
| CL2166 | *dvIs19 [(pAF15)gst-4p::GFP::NLS] III* | 6 | *gst-4* | *dvIs19* [pAF15(gst-4::GFP::NLS] | *wild type* | CL2166 |
| SJ4143 | *zcIs17[ges-1::GFP(mito)]* | 3 | *ges-1* | *zcIs17* [ges-1::GFP(mit)] | *wild type* | SJ4143 |
| VV93 | *unc-26(e205) IV; zcIS17 [ges-1::GFP(mito)]* | 5 | *unc-26* | zcIS17 [ges-1::GFP(mit)] | *e205* | VV78, SJ4143 |
| VV95 | *snt-1(md290) II; zcIS17 [ges-1::GFP(mito)]* | 5 | *snt-1* | *zcIS17 [ges-1::GFP(mit)]* | *md290* | VV80, SJ4143 |
| VV107 | *unc-26(e205) IV; [pAF15(gst-4::GFP::NLS]* | 5 | *unc-26* | *dvIs19*  *[pAF15(gst-4::GFP::NLS]* | *e205* | VV78, CL2166 |
| VV109 | *snt-1(md290) II; [pAF15(gst-4::GFP::NLS]* | 5 | *snt-1* | *dvIs19*  *[pAF15(gst-4::GFP::NLS]* | *md290* | VV80, CL2166 |
| VV114 | *dgk-1(ok1462) X; zcIS17 [ges-1::GFP(mito)]* | 5 | *dgk-1* | *zcIS17 [ges-1::GFP(mit)]* | *ok1462* | VV71, SJ4143 |
| VV116 | *dgk-1(ok1462) X; [pAF15(gst-4::GFP::NLS]* | 5 | *dgk-1* | *dvIs19*  *[pAF15(gst-4::GFP::NLS]* | *ok1462* | VV71, CL2166 |

**Lifespan assay and analysis**

Lifespan assays were conducted in 96-well plates as described in ([Solis and Petrascheck, 2011](#_ENREF_39)). Briefly, age-synchronized animals were cultured in S-complete media containing *E. coli* OP50 as feeding bacteria (~2 × 10^9^ bacteria mL^−1^) in 96-well plates, such that 5–15 worms are in each well. At the L4 stage, FUDR was added to prevent animals from producing offspring. Solvent (water or DMSO) or small molecules were added on day 1 of adulthood, exposing the worms to control or compound treatment until the end of the assay. When used, DMSO was kept to a final concentration of 0.33% v/v. Live animals were scored visually based on movement induced by shaking and application of light to each well. Animals were scored three times a week, until 95% of animals were dead in all the tested conditions. Statistical analysis was performed using the Mantel–Haenszel version of the log-rank test.

**Stress resistance assays**

Resistance to oxidative stress was determined by measuring survival of antidepressant-treated and untreated worms after a 24 h exposure to the ROS generator paraquat (Methyl viologen). Experimental worm cultures were set up as described in Lifespan assays. Solvent (water or DMSO) or antidepressants were added on day 1 of adulthood unless otherwise indicated. For dose response assays, paraquat was added to a final concentration of 0, 25, 50, 75, 100 or 150 mM on day 5 of adulthood. For all experiments with paraquat dose response, survival of worms was assessed 24 h after paraquat addition and expressed as the percentage of live versus total animals. For the serotonin antagonists experiment, heat map and hierarchical clustering analysis (dendrograms) were generated with R using the heatmap.2 function of the gplots package. Dissimilarity between values and hierarchical clustering were calculated using the Euclidean distance measure and complete linkage method.

***In vitro* free radical scavenging assays**

Free radical scavenging activity of antidepressants was measured in a cell-free assay using DPPH as a free radical and Trolox as a positive control scavenger. The DPPH radical absorbs light at 517 nm, while its reduced form does not, allowing to monitor scavenging activity ([Pisoschi et al., 2009](#_ENREF_33)). Mianserin, Mirtazapine and Trolox stock solutions (10 x) were prepared in ethanol, and tested for scavenging activity at 0.045, 0.18, 0.9, 1.8, 9, 18, 90, 900, and 1800 µM using ethanol as a negative control. The assay was carried out in black 96-well plates with clear, flat bottoms testing each condition in triplicates. Twenty µl of antidepressant stock solution was mixed with 180 µl of 100 µM DPPH stock solution (also in ethanol, final conc. 90 µM) and incubated for 30 min in the dark (room temperature). DPPH concentrations were determined by measuring absorbance at 520 nm. Averages between triplicates were calculated and subtracted from the blank. The absorbance of DPPH in ethanol control wells was set as 100%. All values were plotted as DPPH [%] against dual X-axes of log [10] concentration of drug and log [10] ratio of small molecule to DPPH.

**Aldicarb assay**

Synaptic transmission in response to the antidepressants Mianserin and Fluoxetine was measured by determining onset of paralysis by the acetycholine esterase inhibitor aldicarb. For each condition 60-80 L4 stage animals were treated with water, Mianserin (50 µM) or Fluoxetine (100 µM) followed by aldicarb addition (4mM final conc.) 2 h later. Paralysis of animals was scored visually, every 15 min. Results were graphed as the paralyzed fraction of animals [%] as a function of time. The mean paralysis [%] from three independent experiments was calculated and statistical significance was determined using Student’s *t-test*.

**Real-time quantitative PCR (qPCR) and data analysis**

All qPCR experiments were conducted according to the MIQE guidelines ([Bustin et al., 2009](#_ENREF_9)), except that samples were not tested in a bio-analyzer but photometrically quantified using a Nanodrop. All strains were cultured in 96-well plates as described in ([Solis and Petrascheck, 2011](#_ENREF_39)). Water or Mianserin 50µM were added on day 1 of adulthood and worms were harvested on day 5. RNA was extracted as described above, followed by DNAse (Sigma, cat # AMPD1-1KT) treatment and reverse transcription using iScript RT-Supermix (BIO-RAD, cat # 170-8841) at 42ºC for 30 min . Quantitative PCR reactions were set up in 384-well plates (BIO-RAD, cat # HSP3901) and included 2.5 µl Bio-Rad SsoAdvanced SYBR Green Supermix (cat # 172-5264) or Kapa SYBR Fast master mix (cat # KK4602), 1 µl cDNA template (2.5 ng/µl, to final of 0.5 ng/µl in 5 µl PCR reaction), 1 µl water, and 0.5 µl of forward and reverse primers (150 nM final concentration for BIO-RAD SYBR mix and 75 nM final for Kapa SYBR mix) (see Table below for oligo pairs used for qPCR of genes tested). Quantitative PCR was carried out using a BIO-RAD CFX384 Real-Time thermocycler (95ºC, 3 min; 40 cycles of 95ºC 10 s, 60ºC 30 s; Melting curve: 95ºC 5 s, 60ºC- 95ºC at 0.5ºC increment, 10 s). Gene expression was normalized to three reference genes, *rcq-5*, *crn-3* and *rpl-6,* using the BIO-RAD CFX Manager software. Statistical significance was determined using Student’s *t-test*.

**List of oligos used for qPCR**

| **Gene name** | **qPCR forward primer (5’-3’)** | **qPCR reverse primer (5’-3’)** |
| --- | --- | --- |
| sod-1 | CGTAGGCGATCTAGGAAATGTG | AACAACCATAGATCGGCCAACG |
| prdx-2 | CATTCCAGTTCTCGCTGAC | ATGATGAAGAGTCCACGGA |
| crn-3 | GAATGCACTCATGAACAAAGTC | TAATGTTCGACTGATGAACCG |
| rcq-5 | GATGTTAGAGCTGTAATTCACTGG | ATCTCTTCCAGCTCTTCCG |
| rpl-6 | TTCACCAAGGACACTAGCG | GACAGTCTTGGAATGTCCGA |

**Immunoblotting**

Immunoblots were conducted to verify changes in GFP expression on day 5. Worms were cultured in 96-well plates, as described in “lifespan analysis,” treated with Mianserin (50 µM) or water on day 1 of adulthood, and harvested on day 5. Harvested worms were washed with Dulbecco’s phosphate buffer and frozen in liquid nitrogen. Protein was harvested by adding 100 µl lysis buffer (1 mM EDTA, 1 mM EGTA, 1 mM TCEP, protease inhibitors in PBS), zirconium beads and glass beads (cat # 03961-1-103 and cat # 03961-1-104) were added to a ratio of 5:1:1, respectively and disrupted in Precellys lysing system (6500 rpm, 3 x 10 s cycles). For each condition, worms from at least 48 wells (~500 worms) were pooled together, per biological replicate. Protein concentrations were measured using the Bradford assay (BIORAD). Equal amounts of protein (20 μg/lane) were separated on 12.5% SDS-polyacrylamide gels under reducing conditions and proteins were transferred to nitrocellulose membranes (BIO-RAD, cat # 162-0112). Membranes were blocked in 5% non-fat milk in PBS and incubated overnight in 5% BSA containing rabbit anti-GFP primary antibody (Genetex, cat # GTX113617) at 1:2000 dilution. After washing excess non-bound antibody with PBS-T, goat anti-rabbit IRdye 800CW (LI-COR, cat # 827-08365) was added for 1 h at room temperature. After washing, bound antibodies were visualized and imaged using LI-COR IR dye detection system. For loading control, the membrane was blocked with 5% milk followed by mouse anti-actin (MP Biomedicals, cat # 691001) in 1:10,000 dilution in 1% BSA for 1 h. After washing, membrane was incubated with secondary antibody goat anti-mouse IRdye 800CW (LI-COR, cat # 827-08364) for 1 h, washed and imaged using LI-COR Odyssey IR dye detection system. The GFP band intensity was quantified and normalized to that of actin using LI-COR Odyssey quantification software tool. Data from independent experiments were analyzed and statistical significance was determined using Student’s *t-test*.

**Fluorescent microscopy, imaging, and quantification**

Reporter strains were cultured in 96-well plates ([Rangaraju et al., 2015](#_ENREF_34); [Solis and Petrascheck, 2011](#_ENREF_39)) and exposed to compounds on day 1 of adulthood. Concentrations varied dependent on compound: Mianserin was used at 0, 2, 10, 50, 100 or 200 µM; Fluoxetine was used at 0, 50, 100, 250 or 500 µM; paraquat was used at 100 mM. Fluorescent images were taken 24 h after Mianserin or Fluoxetine treatment, and 8 h after paraquat treatment. For imaging, animals were washed several times to remove eggs and bacteria using paralysis solution (0.3 mg/ml levamisole, 0.005% Triton X-100 in M9), pooled (8-16 wells per condition ~100-150 worms) and transferred into a well of a black, clear-bottom imaging plate ([Shore et al., 2012](#_ENREF_37)). Bright field and fluorescent images were taken by a Molecular Devices ImageXpress platform. Fluorescence for each worm was quantified by the Cell Profiler software using the bright-field image to create a mask for each worm used to measure the mean fluorescence per area after median background subtraction. Statistical significance was determined using *t-test*.
